# Heart Failure Care in Low- and Middle-Income Countries: A Systematic Review and Meta-Analysis

**DOI:** 10.1371/journal.pmed.1001699

**Published:** 2014-08-12

**Authors:** Thomas Callender, Mark Woodward, Gregory Roth, Farshad Farzadfar, Jean-Christophe Lemarie, Stéphanie Gicquel, John Atherton, Shadi Rahimzadeh, Mehdi Ghaziani, Maaz Shaikh, Derrick Bennett, Anushka Patel, Carolyn S. P. Lam, Karen Sliwa, Antonio Barretto, Bambang Budi Siswanto, Alejandro Diaz, Daniel Herpin, Henry Krum, Thomas Eliasz, Anna Forbes, Alastair Kiszely, Rajit Khosla, Tatjana Petrinic, Devarsetty Praveen, Roohi Shrivastava, Du Xin, Stephen MacMahon, John McMurray, Kazem Rahimi

**Affiliations:** 1 The George Institute for Global Health, University of Oxford, Oxford, United Kingdom; 2 The George Institute for Global Health, University of Sydney, Sydney, Australia; 3 Institute for Health Metrics and Evaluation, University of Washington, Seattle, Washington, United States of America; 4 Non-Communicable Diseases Research Centre, Tehran University of Medical Sciences, Tehran, Iran; 5 Endocrinology and Metabolism Research Centre, Tehran University of Medical Sciences, Tehran, Iran; 6 Effi-Stat, Paris, France; 7 Department of Cardiology, Royal Brisbane and Women's Children Hospital and University of Queensland School of Medicine, Brisbane, Australia; 8 Department of Epidemiology, Shahid Beheshti University of Medical Sciences, Tehran, Iran; 9 The George Institute for Global Health, Hyderabad, India; 10 Clinical Trials Service Unit, University of Oxford, Oxford, United Kingdom; 11 National University of Singapore, Singapore; 12 Hatter Institute for Cardiovascular Research in Africa, University of Cape Town, Cape Town, South Africa; 13 Faculdade de Medicina da Universidade de São Paulo, São Paulo, Brazil; 14 National Cardiovascular Centre University Indonesia, Jakarta, Indonesia; 15 Universidad Nacional del Centro de la Provincia de Buenos Aires, Buenos Aires, Argentina; 16 Centre Hospitalier Universitaire de Poitiers, Poitiers Cedex, France; 17 Centre of Cardiovascular Research & Education in Therapeutics, Monash University, Melbourne, Australia; 18 Bodleian Healthcare Libraries, University of Oxford, Oxford, United Kingdom; 19 The George Institute for Global Health, Peking University, Beijing, China; 20 University of Glasgow, Glasgow, United Kingdom; Umeå Centre for Global Health Research, Umeå University, Sweden

## Abstract

**Background:**

Heart failure places a significant burden on patients and health systems in high-income countries. However, information about its burden in low- and middle-income countries (LMICs) is scant. We thus set out to review both published and unpublished information on the presentation, causes, management, and outcomes of heart failure in LMICs.

**Methods and Findings:**

Medline, Embase, Global Health Database, and World Health Organization regional databases were searched for studies from LMICs published between 1 January 1995 and 30 March 2014. Additional unpublished data were requested from investigators and international heart failure experts. We identified 42 studies that provided relevant information on acute hospital care (25 LMICs; 232,550 patients) and 11 studies on the management of chronic heart failure in primary care or outpatient settings (14 LMICs; 5,358 patients). The mean age of patients studied ranged from 42 y in Cameroon and Ghana to 75 y in Argentina, and mean age in studies largely correlated with the human development index of the country in which they were conducted (*r* = 0.71, *p*<0.001). Overall, ischaemic heart disease was the main reported cause of heart failure in all regions except Africa and the Americas, where hypertension was predominant. Taking both those managed acutely in hospital and those in non-acute outpatient or community settings together, 57% (95% confidence interval [CI]: 49%–64%) of patients were treated with angiotensin-converting enzyme inhibitors, 34% (95% CI: 28%–41%) with beta-blockers, and 32% (95% CI: 25%–39%) with mineralocorticoid receptor antagonists. Mean inpatient stay was 10 d, ranging from 3 d in India to 23 d in China. Acute heart failure accounted for 2.2% (range: 0.3%–7.7%) of total hospital admissions, and mean in-hospital mortality was 8% (95% CI: 6%–10%). There was substantial variation between studies (*p*<0.001 across all variables), and most data were from urban tertiary referral centres. Only one population-based study assessing incidence and/or prevalence of heart failure was identified.

**Conclusions:**

The presentation, underlying causes, management, and outcomes of heart failure vary substantially across LMICs. On average, the use of evidence-based medications tends to be suboptimal. Better strategies for heart failure surveillance and management in LMICs are needed.

*Please see later in the article for the Editors' Summary*

## Abstract 2

In a systematic review and meta-analysis, Kazem Rahimi and colleagues examine the burden of heart failure in low- and middle-income countries.

*Please see later in the article for the Editors' Summary*

## Editors' Summary

**Background:**

A healthy heart pumps about 23,000 liters of blood around the body every day. This blood delivers oxygen and nutrients to the rest of the body and carries carbon dioxide and waste products away from the tissues and organs. A healthy heart is therefore essential for life. Unfortunately, many people (particularly elderly people) develop heart failure, a life-threatening condition in which the heart no longer pumps enough blood to meet all the body's needs because it has become too weak or too stiff to work properly. Heart failure can affect the left, right, or both sides of the heart, and it can develop slowly (chronic heart failure) or quickly (acute heart failure). Its symptoms include swelling (edema) of the feet, ankles, and legs, tiredness, and shortness of breath. Heart failure, which is most commonly caused by coronary heart disease (blockage with fatty deposits of the blood vessels that supply the heart) or high blood pressure (hypertension), cannot be cured. However, lifestyle changes (for example, losing weight and avoiding salty food) and various medications can control heart failure and improve the quality of life of patients.

**Why Was This Study Done?:**

In high-income countries (HICs), heart failure is a common condition that typically consumes 1%–2% of healthcare resources. Experts believe that heart failure may soon become a major public health issue in low- and middle-income countries (LMICs) because fewer people are dying of infectious diseases in these countries than in the past. LMICs need to plan for this eventuality, but little is known about the current burden of heart failure in LMICs. Here, the researchers undertake a systematic review and meta-analysis of published and unpublished information on the presentation, causes, management, and outcomes of heart failure in LMICs. A systematic review uses predefined criteria to identify all the research on a given topic; a meta-analysis uses statistical approaches to combine the results of several studies.

**What Did the Researchers Do and Find?:**

The researchers identified 49 published studies and four unpublished databases that provided information on nearly 240,000 hospitalizations for acute and chronic heart failure in 31 LMICs. Across these LMICs, the average age of patients admitted to hospital for heart failure was 63 years, more than ten years younger than the average admission age in HICs. Differences in mean age at presentation, which ranged from 42 years in Cameroon and Ghana to 75 years in Argentina, largely correlated with the human development index (a measure of national well-being) of individual LMICs. Notably, acute heart failure accounted for 2.2% of all hospital admissions in the LMICs for which data were available. Hypertension was the main cause of heart failure in Africa and the Americas, whereas ischemic heart disease was the main cause in all other regions. More than two-thirds of patients were prescribed diuretics for heart failure, whereas only 57% of patients were treated with angiotensin-converting enzyme inhibitors, only 34% were treated with beta-blockers, and only 32% were treated with mineralocorticoid receptor antagonists, the three treatments currently recommended in guidelines for managing heart failure. Finally, on average, patients admitted to hospital for heart failure in LMICs spent ten days in hospital, and 8.3% of them died in hospital (compared to 6.7% and 4% of similar patients across Europe and the US, respectively).

**What Do These Findings Mean?:**

These findings show that the presentation, causes, management, and outcomes of heart failure vary substantially across LMICs. Importantly, however, these findings reveal that heart failure is already a major burden in LMICs and that the use of recommended medications for heart failure is currently suboptimal in these countries. Because the studies included in this systematic review and meta-analysis set out to answer different research questions and used different methods to diagnose heart failure, the estimates of the burden of heart failure in LMICs provided here may not be completely accurate. Moreover, because the data were derived mainly from urban tertiary referral hospitals, these findings may not reflect the broader picture of heart failure in the community in LMICs. However, although additional studies are needed to completely assess the burden of heart failure in LMICs, the present findings nevertheless highlight the need to implement better strategies for the management of heart failure in LMICs.

**Additional Information:**

Please access these websites via the online version of this summary at http://dx.doi.org/10.1371/journal.pmed.1001699.

- This study is further discussed in a *PLOS Medicine* Perspective by Druin Burch
- The US National Heart, Lung, and Blood Institute provides information for patients about heart failure
- The UK National Health Service Choices website provides information about all aspects of heart failure
- The American Heart Association, a not-for-profit organization, also provides detailed information about heart failure for patients and their carers
- The British Heart Foundation, a not-for-profit organization, provides additional information about heart failure, including a personal story about heart failure; further personal stories about heart failure are provided by the not-for-profit organization Healthtalkonline
- Heart Failure Matters provides practical information about heart failure for patients, families, and caregivers in several languages; its website includes an animated journey through heart failure and several personal stories about the condition
- MedlinePlus provides links to further resources about heart failure (in English and Spanish)

## Introduction

In high-income countries (HICs), heart failure is a well-recognized public health problem representing a significant burden for patients and healthcare systems [1],[2]. For example, in the UK and US, heart failure is one of the leading causes of hospitalisation, and despite recent advances, outcomes remain poor [3]–[6]. Of those hospitalised for heart failure in the UK, about 10% will die during admission [6]. In the US, between 20% and 27% of those who survive to discharge will be re-admitted within 30 d [7], whilst 5-y mortality rates range between 40% and 65% amongst the US, UK, Netherlands, and Sweden [2]–[4],[8],[9]. The costs associated with heart failure care are also substantial. In many HICs, heart failure typically consumes 1%–2% of healthcare resources [2], mainly because of repeated admissions to hospitals and prolonged inpatient stays.

With demographic changes and the epidemiological transition to non-communicable diseases [10],[11], heart failure is expected to become a major public health issue in low- and middle-income countries (LMICs). Yet systematic evidence for its current burden to patients and health services is limited [1],[2],[12]. In fact, the last review of the burden of heart failure in LMICs, conducted over ten years ago, found no population studies and concluded that published data on heart failure epidemiology were almost entirely absent from most populations across the world [12]. As a result, many of our assumptions regarding the current burden of this condition worldwide are based on extrapolations from studies conducted in HICs, which may not be appropriate [1],[2].

Therefore, we sought to conduct a systematic review of both published and unpublished data regarding the patterns of heart failure presentation, management, and outcomes in LMICs.

## Methods

This systematic review was designed and undertaken according to the Preferred Reporting Items for Systematic Reviews and Meta-Analyses (PRISMA) guidelines [13]. A study protocol describing the methodology has been published previously [14]. In brief, we searched Medline, Embase, Global Health Database, and WHO regional databases for articles published between 1 January 1995 and 30 March 2014 with the subject terms “heart failure” or “cardiomyopathies” or any related terms AND “incidence”, “prevalence”, “cause*”, “etiology”, “aetiology”, “epidemiolog*”, “burden”, “management”, “treatment”, “prevent*”, “population based”, “community”, “trends”, “survey”, “surveillance”, “mortality”, “morbidity”, “fatalit*”, or “attack rate”. Relevant studies from LMICs on the epidemiology, diagnosis, management, and outcomes of heart failure were included. There were no language restrictions. We also scrutinised the reference lists of study reports and review articles, and inquired among our collaborators and international heart failure experts about any additional databases or studies of which they may be aware. We further searched the Institute for Health Metrics and Evaluation's Global Health Data Exchange as well as the websites of regional and country-specific societies of cardiology to identify further datasets.

**Figure 1:**
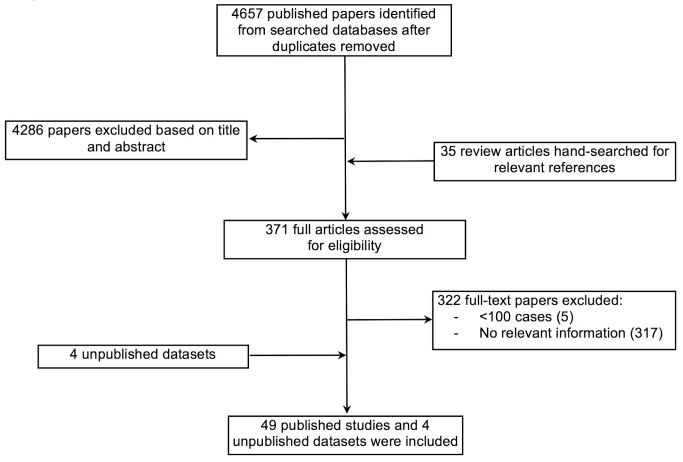
Data acquisition flowchart.

Figure 1 summarises the retrieval and selection process for studies and relevant databases. After removing duplicate reports, two reviewers independently screened all titles and abstracts for their potential eligibility and extracted data using a pre-designed form. Studies were eligible for inclusion if they reported on heart failure patients from LMICs as defined by the World Bank [15]. Studies must have reported on at least 100 cases and contained relevant information on demographic characteristics, prevalence, case fatality, underlying aetiology, or management of patients with heart failure. Studies confined to subgroups of patients with heart failure (for example, those that included only dilated cardiomyopathy or heart failure as a complication of acute myocardial infarction) were excluded, as were studies that clearly did not include a representative sample of patients from the setting chosen (for example, studies that selected people referred to an echocardiography department, or studies that excluded adult populations) [14]. Investigators of multinational studies that had not reported findings by country were contacted for country-specific data.

### Quality Assessment

In order to capture a comprehensive overview of heart failure in LMICs, a wide range of studies, each with differing objectives and designs, were included. Studies meeting the minimum quality requirement, as specified below, for inclusion were analysed for both methodological limitations and reporting quality, using items from the Strengthening the Reporting of Observational Studies in Epidemiology (STROBE) guidelines [16] (Tables 1–6). Specifically, the sample size of each study, the location and type of healthcare facility, diagnostic methods used, and patient selection criteria were documented. In addition, we assessed each study's specific methodological strengths and weaknesses as well as likely external validity.

**Table 1:** Characteristics of Africa region studies and databases included.

Country of Origin	Study Design	Recruitment Period	Selection Criteria	Heart Failure Definition	Cases of Heart Failure	Strengths and Limitations
Algeria [70] ∧	Prospective	2008–2009	All outpatients ≥21 y of age with either a previous or new diagnosis of heart failure.Exclusion: patients with acute decompensated heart failure, or those in another clinic trial.	Clinical diagnosis on the basis of the Framingham criteria. [71].91% of participants had an echocardiogram.	400	These data come from the I PREFER registry.Strengths: >10 centres. Sites were randomly selected, and all cardiologists within the country considered eligible. Missing data and loss to follow-up transparent. Prospective trial within a specified recruitment period. >90% had confirmation of heart failure through echocardiography.Limitations: Representative only of those attending outpatient cardiology services, excluding the acute sector or those patients in primary care with heart failure not under joint care of a cardiologist.
Cameroon [24]	Prospective and retrospective elements	1998–2001	Consecutive patients ≥15 y of age admitted to the cardiology clinic and/or the medical wards of Yaounde General Hospital. Those who had not had an echocardiogram were excluded.A prospective phase was carried out between September and November 2001, where all patients with suspected heart failure were included (39 patients).A retrospective phase involved the use of case notes of those with heart failure admitted to the hospital and undergoing echocardiography between 1998 and September 2001 (128 patients).	Clinical diagnosis on the basis of the Framingham criteria [71].All patients had an echocardiogram.	167	Strengths: All patients had echocardiographic assessment.Limitations: This is a study of a single regional tertiary referral centre set in a rural area that may not be representative of the broader population. Patients who had not had an echocardiogram were excluded, but it is unclear how many patients with a clinical diagnosis of heart failure were thus excluded and to what extent this reduces the generalizability of the study findings. Missing data unreported.
Cameroon [29]	Prospective	2002–2008	All consecutive patients diagnosed with congestive cardiac failure referred to the cardiac centre of St. Elizabeth Catholic General Hospital, Shisong, Cameroon.	Clinical diagnosis on the basis of the Framingham criteria.Echocardiography used, but no indication if all patients underwent this investigation.	462	Strengths: Comprehensive prospective study encompassing all patients diagnosed within the study period. Loss to follow-up documented.Limitations: This is a study of a single regional cardiology referral centre that may not be representative of the broader population. Missing data not transparently accounted for.
Democratic Republic of the Congo [32]	Prospective	2003–2004	Every fourth patient admitted with heart failure as an inpatient having been seen at the cardiology clinic of the Lomo Medical Centre of the Heart of Africa Cardiovascular Centre in Kinshasa.	Echocardiography.	100	Strengths: All patients had echocardiographic assessment.Limitations: This is a study of a single urban outpatient cardiology referral centre that may not be representative of the broader population. Missing data not transparently accounted for.
Ghana [30]	Prospective	1992–1995	Consecutive patients with heart failure referred to the National Cardiothoracic Centre, Accra, over 4 y.	Framingham criteria.All patients had an echocardiogram performed.	572	Strengths: This centre receives referrals from all hospitals across the country, increasing the generalizability of the results. All patients had echocardiography.Limitations: Acknowledged potential for referral bias as patients at this single urban tertiary specialist centre may not be representative of heart failure management elsewhere. Unclear if there were missing data, and how they were accounted for.
Nigeria [36]	Retrospective	1995–2005	The case notes of 202 patients with heart failure were randomly selected from the outpatient and inpatient departments of University College Hospital, Ibadan.	New York Heart Association classification.	202	Limitations: Retrospective study with uncertain diagnostic accuracy. Inpatient and outpatient management were not separated. This is a study of a single urban tertiary referral centre that may not be representative of the broader population. Missing data not transparently accounted for.
Nigeria [34]	Retrospective	1996–2005	All patients recorded as having a diagnosis of heart failure from the mortality records of the University of Ilorin Teaching Hospital	Not specified.	228	Strengths: Comprehensive review of all deaths and their respective case notes from the hospital, limiting selection bias.Limitations: Uncertain diagnostic accuracy. This is a single urban teaching hospital providing services to north-central Nigeria. Although the hospital covers a large catchment area, the patients may nonetheless not be representative of the broader population.
Nigeria [33]	Prospective	1997–2001	Records of all patients admitted with cardiovascular disease to the Obafemi Awolowo University Teaching Hospitals Complex in Ife, Nigeria.	Not specified.	386	Strengths: Single tertiary referral centre providing services to 10 million individuals in the southwest of Nigeria, increasing the study's generalizability.Limitations: Single centre, though with a large catchment area, may not be representative of the broader population. No standardised diagnostic criteria used.
Nigeria [37]	Retrospective	1998–2001	All patients admitted to the medical wards of the University of Uyo Teaching Hospital in southern Nigeria with heart failure during the dry seasons within the study period.Exclusion: Patients with renal disease or suspected coronary artery disease.	Clinical features with the aid of blood results, chest radiography, electrocardiography, and echocardiography. The proportion receiving additional investigations is unknown.	245	Strengths: Comprehensive assessment of patients with heart failure as coded for by this hospital.Limitations: Single tertiary referral centre may not be representative of the broader population. Study was a retrospective study of case notes; consequently, diagnostic accuracy is uncertain. Proportions receiving additional gold-standard investigations, such as echocardiography, not documented.
Nigeria [31]	Retrospective	2001–2005	All adults ≥18 y with congestive cardiac failure admitted to the medical wards of the University of Port Harcourt Teaching Hospital.Exclusion: patients whose condition did not meet the Framingham criteria or who died within 24 h of admission.	Framingham criteria.	423	Strengths: Clear diagnostic criteria. Comprehensive assessment of patients with heart failure.Limitations: Single tertiary referral centre may not be representative of the broader population. Retrospective assessment with uncertain accuracy of the aetiology of heart failure. Unclear what proportion received additional investigations such as echocardiography. Unclear how missing data were accounted for.
Nigeria [22]	Prospective	May–June 2004	Consecutive patients ≥18 y with suspected heart failure presenting to outpatient department, wards, or the casualty unit of Jos University Teaching Hospital.	Framingham criteria.	102	Strengths: Consecutive patients included, limiting potential for selection bias. Clear documentation of rationale behind sample size. Standardised diagnosis criteria used. Acknowledged limitations.Limitations: Single tertiary referral centre may not be representative of the broader population. Inpatient and outpatient sample not separated. Aetiology of heart failure ascertained by case notes and clinical findings on examination rather than gold-standard investigation. Echocardiography not available to all patients.
Nigeria [26]	Prospective	2006–2008	Consecutive patients ≥15 y with heart failure presenting to the University of Abuja Teaching Hospital.	European Society of Cardiology guidelines.Echocardiography available for all patients.	340	Strengths: Large catchment area for this referral centre, improving generalizability. All patients had echocardiographic assessment, improving overall diagnostic accuracy and that of assigned underlying aetiologies of heart failure.Limitations: Single tertiary referral centre may reflect more severe cases or those of uncertain diagnosis, therefore not reflecting practice in the broader health service.
Nigeria [27]	Prospective	2006–2010	Clinical registry of consecutive individuals referred for the first time to the cardiology clinic of the University of Abuja Teaching Hospital.Exclusion: those with musculoskeletal chest pain or hepatic or renal failure.	European Society of Cardiology guidelines.Echocardiography available from >95% of patients.	475	Strengths: Consecutive patients, reducing the risk of selection bias. Clear, standardised, diagnostic criteria. Documented use of the STROBE guidelines [16] for the reporting of observational studies.Limitations: Single tertiary referral centre may reflect more severe cases or those of uncertain diagnosis, therefore not reflecting practice in the broader health service.
Nigeria [28]	Prospective	Unknown (published 2009)	177 consecutive individuals with heart failure presenting to the University College Hospital, Ibadan.	Framingham criteria.All patients underwent an echocardiogram.	177	Strengths: Clear, standardised, diagnostic criteria. All patients had an echocardiogram, improving the accuracy of heart failure diagnosis and that of underlying aetiology. Catchment area of greater than 3 million individuals, improving the generalizability of the results. Clear explanation of statistical methods used.Limitations: Single tertiary referral centre may reflect more severe cases or those of uncertain diagnosis, therefore not reflecting practice in the broader health service.
Senegal [38]	Prospective	January–June 2001	Selection criteria not specified. Urban hospital in Dakar.	Clinical diagnosis.All patients underwent echocardiography.	170	Strengths: All patients underwent echocardiography, improving the likely accuracy of the diagnosis of heart failure and of assigned aetiology.Limitations: Single urban hospital in the capital may not reflect broader population with heart failure. Unclear selection criteria.
South Africa [25]	Prospective	2006	All patients with cardiovascular disease or presenting to the cardiology unit. Those with a de novo presentation with heart failure were included.Exclusion: those with acute ischaemic aetiology.	Based on European Society of Cardiology guidelines.All patients had an echocardiogram.	844	Strengths: Sole cardiovascular centre for a population of 1.1 million individuals, increasing the generalizability of findings. All patients underwent echocardiographic assessment, improving likely accuracy of diagnosis and of underlying aetiology of each patient's heart failure. Clear documentation of data availability and criteria applied.Limitations: Exclusion of those with an ischaemic aetiology may underestimate the proportion of those with heart failure due to IHD. Urban hospital setting may not reflect the broader population.
Sub-Saharan Africa [35]	Prospective	2007–2010	Patients ≥12 y with acute heart failure confirmed by echocardiography were included.The study was conducted in the following countries: Sudan, Ethiopia, Kenya, Uganda, Mozambique, South Africa, Cameroon, Nigeria, Senegal.Exclusion: those with acute ST-elevation myocardial infarction, known severe renal failure, hepatic failure, or another cause of hypoalbuminemia.	Unspecified signs and symptoms of heart failure.All patients had an echocardiogram.	1,006	Strengths: All patients had echocardiographic assessment, improving diagnostic accuracy. Clear documentation of missing data and loss to follow-up as well as how this was accounted for in analyses. First published data on heart failure from a number of African countries.Limitations: Urban single hospital centres included. Individual study sites often had very few patients enrolled (range from 10 to 200). Exclusion criteria may lead to the underestimation of IHD as a cause of heart failure.

∧ Previously unpublished data.

**Table 2:** Characteristics of Americas region studies and databases included.

Country of Origin	Study Design	Recruitment Period	Selection Criteria	Heart Failure Definition	Cases of Heart Failure	Strengths and Limitations
Argentina [23]	Retrospective	1992–1999	All patients diagnosed with congestive heart failure, decompensated heart failure, or acute pulmonary oedema as recorded in the electronic vital statistics of a community hospital of Mar del Plata, Argentina.	Not specified.	6,368	Strengths: Comprehensive study with limited selection bias.Limitations: Single community hospital that may not be reflective of broader patterns of heart failure prevalence. No standardised method for diagnosing heart failure, relying on discharge reports.
Argentina [21]	Prospective	1996–1997	Patients admitted to both the general medical and cardiology wards with decompensated chronic heart failure. Patients must have had heart failure, as diagnosed by the Framingham clinical criteria, for 30 d or more.Exclusion: acute heart failure due to an ischaemic event, those lost to follow-up, and those without an electrocardiogram and chest radiograph.	Framingham criteria.Unspecified proportion received echocardiography.	751	Strengths: 31 centres from across Argentina, 42% of which were in Buenos Aires. Standardised diagnostic criteria. Clear statistical methods documented.Limitations: Centres were invited to take part rather than randomised. Uncertain proportion received echocardiographic confirmation. Exclusion criteria may lead to underestimation of IHD as an aetiology of heart failure.
Argentina [43]	Prospective	2002–2003	All patients >18 y hospitalised for decompensated chronic heart failure.Exclusion: heart failure secondary to a myocardial infarction or post-operatively.	Investigator's discretion.	615	Strengths: 36 centres predominantly based around Buenos Aires or neighbouring regions. Comprehensive assessment of all patients with likely low selection bias.Limitations: Centres were not randomised, rather invited. Consequently, results may not reflect the broader management of heart failure amongst physicians with less of an interest in heart failure. No standard diagnostic criteria. Exclusion criteria may lead to underestimation of IHD as an aetiology of heart failure. Uncertain adjustment for those with missing data.
Argentina [44]	Prospective	2007	All patients >18 y of age were included if hospitalised for decompensated chronic heart failure.Exclusion: heart failure as a complication of a myocardial infarction or post-operatively.	Investigator's discretion.	736	Strengths: 36 centres from across Argentina.Limitations: Centres invited to take part rather than randomised, and those that did may reflect clinicians with an interest in heart failure, affecting the broader generalizability of results. Exclusion criteria may lead to underestimation of IHD as an aetiology of heart failure. No standard diagnostic criteria. Uncertain adjustment for those with missing data or lost to follow-up. No standard diagnostic criteria for heart failure.
Brazil [45]	Retrospective	1992 to 2010	Patients admitted to public hospitals in São Paulo with heart failure.	Not specified.	194,098	Strengths: From the Datasus registry, providing hospital episode statistics for the entire public health system of São Paulo.Limitations: Uncertain diagnostic criteria based on individual physician's discretion.
Brazil [42]	Prospective	1998–2000	Consecutive patients admitted to hospital with worsening symptoms of heart failure (NYHA functional classes III or IV).Exclusion: patients with heart failure due to valvular heart diseases, thyrotoxicosis, hypothyroidism, severe anaemia, amyloidosis, neoplasia, chronic non-cardiogenic pulmonary diseases, previous heart transplantation, chronic haemodialysis, or participation in drug protocols.	Clinical diagnosis based on the Framingham criteria.	494	Strengths: Standardised diagnostic criteria.Limitations: University Teaching Hospital in São Paulo dedicated to cardiology. Exclusion criteria may further hinder generalizability. Only patients with NYHA functional class III or IV, so may not be generalizable to those with milder symptoms. The exclusion of patients with valvular heart disease may impact on the assignment of aetiologies of heart failure. Unclear how loss to follow up and missing data were accounted for.
Brazil [40]	Prospective	2001	98 consecutive patients admitted to participating public hospitals and 105 consecutive patients admitted to participating private hospitals within the 3-mo study period in the city of Niteroi with a Boston criteria score of 8 or more.	Boston criteria score ≥8.	203	Strengths: Multiple hospitals within Niteroi, improving generalizability. Clear statistical methods reported. Just under half of patients were from the private sector, the remaining from the public sector, allowing direct comparison between these two groups and representation from a broader swathe of society.Limitations: The methods used to select the participating hospitals are unclear, as is the final number of sites included.
Brazil [19]	Prospective	2005–2006	Consecutive patients admitted with heart failure and systolic dysfunction.	Clinical diagnosis with echocardiographic confirmation.	263	Limitations: Single urban centre that may not be representative of the patterns of care at the national level. Unclear how missing data and loss to follow-up were accounted for. Uncertain diagnostic criteria or proportion receiving echocardiography. Only patients with systolic dysfunction were included, possibly reducing the generalizability of results.
Brazil [39]	Prospective	2006–2008	Consecutive patients ≥18 y referred to heart failure clinic with a Boston score of ≥7. Individuals were all classed as living in rural areas as per the Brazilian Institute of Geography and Statistics.	Boston criteria score ≥7.All patients underwent echocardiography.	166	Strengths: All patients had echocardiographic assessment. Standard diagnostic criteria.Limitations: Single centre study that may not be representative of the patterns of care at the national level.
Brazil [46]	Prospective	Unknown (published 2008)	Patients consecutively admitted to the emergency department of the Instituto do Coração do Hospital das Clínicas da Faculdade de Medicina da Universidade de São Paulo over a period of 150 d with the diagnosis of decompensated heart failure. 100 out of the 212 patients initially assessed were retrospectively selected, for whom further details were collected.	Not specified.	100	Strengths: Although there were no standard diagnostic criteria for heart failure itself, there were standard criteria for assigning aetiologies of heart failure.Limitations: Single urban tertiary referral centre that may not be representative of the patterns of care at the national level. No standard diagnostic criteria used. Method of selection of the 100 patients for whom more detailed analysis was performed unclear.
Brazil [59]	Prospective	16 unspecified months (published 2012).	Tertiary centre in Salvador, Bahia, Brazil. Consecutive patients with a diagnosis of heart failure who had had echocardiography.	Echocardiography.	383	Strengths: All patients had echocardiographic assessment. Standard criteria for the assignment of aetiologies.Limitations: Single urban tertiary referral centre that may not be representative of the patterns of care at the national level. Only those patients who had already had echocardiography were included. Endemic zone for Chagas disease, which may hinder the generalizability of the study.
Brazil [67]	Retrospective	2008	All patients with congestive heart failure treated at the outpatient clinic of Hospital das Clínicas of the Federal University of Goiás.Exclusion: those who died in 2008 (their medical records were incomplete) or who were not from the state of Goiás.	Not specified.	144	Strengths: Unbiased case selection.Limitations: Retrospective use of case notes without specified diagnostic criteria. Single urban centre that may not be representative of the patterns of care at the national level. Patients who died within the time frame of the study were excluded, limiting the study to patients with less severe forms of heart failure.
Brazil [65]	Prospective	1997	100 patients were randomly selected from the outpatient department of the Hospital das Clinicas, a tertiary referral centre in São Paulo. Patients were included if they were found on echocardiography to have a LVEF of <60%.	Echocardiography.	100	Strengths: All patients had echocardiography performed, aiding with the accuracy of diagnosis.Limitations: Single urban tertiary referral centre that may not be representative of the broader population.
Brazil [66]	Retrospective	1995	Analysis of those patients admitted with heart failure to the Heart Institute of São Paulo using the PRODESP registry.	Not specified.	903	Strengths: Dataset of all patients admitted over the course of 1995 with heart failure to this hospital.Limitations: Specialist heart failure urban hospital, whose patients may not be generalizable. No formal standard for the diagnosis of heart failure is documented.
Chile [41]	Prospective	2002–2004	372 patients with NYHA class III or IV heart failure from 14 centres in Chile were included.Exclusion: principal reason for hospitalisation was not heart failure or new-onset heart failure or cardiogenic shock secondary to a myocardial infarction.	Clinical diagnosis using European Society of Cardiology diagnostic criteria. In cases of doubt response to treatment was used.52% underwent echocardiography.	372	Strengths: National Registry of Heart Failure of Chile, 14 centres. Clear diagnostic criteria.Limitations: Choice of participating centres not described. Exclusion of patients with heart failure after a myocardial infarction may lead to artificially low rates of IHD as the attributed cause of heart failure.
Chile [70] ∧	Prospective	2008–2009	All outpatients ≥21 y of age with new or previously diagnosed heart failure.Exclusion: patients with acute decompensated heart failure.	Framingham criteria.78% had an echocardiogram.	199	These data come from the I PREFER registry.Strengths: >10 centres. Sites were randomly selected, and all cardiologists within the country considered eligible. Missing data and loss to follow-up transparent. Prospective trial within a specified recruitment period. 78% had confirmation of heart failure through echocardiography.Limitations: Representative only of those attending outpatient cardiology services, excluding the acute sector or those patients in primary care with heart failure not under joint care of a cardiologist.
Colombia [70] ∧	Prospective	2008–2009	All outpatients ≥21 y of age with new or previously diagnosed heart failure.Exclusion: patients with acute decompensated heart failure.	Framingham criteria.72% had an echocardiogram.	211	These data come from the I PREFER registry.Strengths: >10 centres. Sites were randomly selected, and all cardiologists within the country considered eligible. Missing data and loss to follow-up transparent. Prospective trial within a specified recruitment period. >70% had confirmation of heart failure through echocardiography.Limitations: Representative only of those attending outpatient cardiology services, excluding the acute sector or those patients in primary care with heart failure not under joint care of a cardiologist.
Mexico [70] ∧	Prospective	2008–2009	All outpatients ≥21 y of age with new or previously diagnosed heart failure.Exclusion: patients with acute decompensated heart failure.	Framingham criteria.75% had an echocardiogram.	458	These data come from the I PREFER registry.Strengths: >10 centres. Sites were randomly selected, and all cardiologists within the country considered eligible. Missing data and loss to follow-up transparent. Prospective trial within a specified recruitment period. 75% had confirmation of heart failure through echocardiography.Limitations: Representative only of those attending outpatient cardiology services, excluding the acute sector or those patients in primary care with heart failure not under joint care of a cardiologist.

∧ Previously unpublished data.

NYHA, New York Heart Association.

**Table 3:** Characteristics of Eastern Mediterranean region studies and databases included.

Country of Origin	Study Design	Recruitment Period	Selection Criteria	Heart Failure Definition	Cases of Heart Failure	Strengths and Limitations
Egypt [70] ∧	Prospective	2008–2009	All outpatients ≥21 y of age with new or previously diagnosed heart failure.Exclusion: patients with acute decompensated heart failure.	Framingham criteria.73% had an echocardiogram.	434	These data come from the I PREFER registry.Strengths: >10 centres. Sites were randomly selected, and all cardiologists within the country considered eligible. Missing data and loss to follow-up transparent. Prospective trial within a specified recruitment period. >90% had confirmation of heart failure through echocardiography.Limitations: Representative only of those attending outpatient cardiology services, excluding the acute sector or those patients in primary care with heart failure not under joint care of a cardiologist.
Iran#,∧	Retrospective	1998–2012	All 277 patients with heart failure from a dataset of 83,895 hospitalised patients in Iran.Unpublished dataset.	Not specified.	277	Strengths: Multi-centre study.Limitations: Non-random selection of hospitals. Diagnostic criteria used unspecified.
Iran [70] ∧	Prospective	2008–2009	All outpatients ≥21 y of age with new or previously diagnosed heart failure.Exclusion: patients with acute decompensated heart failure.	Framingham criteria.95% had an echocardiogram.	105	These data come from the I PREFER registry.Strengths: >10 centres. Sites were randomly selected, and all cardiologists within the country considered eligible. Missing data and loss to follow-up transparent. Prospective trial within a specified recruitment period. >90% had confirmation of heart failure through echocardiography.Limitations: Representative only of those attending outpatient cardiology services, excluding the acute sector or those patients in primary care with heart failure not under joint care of a cardiologist.
Lebanon [70] ∧	Prospective	2008–2009	All outpatients ≥21 y of age with new or previously diagnosed heart failure.Exclusion: patients with acute decompensated heart failure.	Framingham criteria.83% had an echocardiogram.	181	These data come from the I PREFER registry.Strengths: >10 centres. Sites were randomly selected, and all cardiologists within the country considered eligible. Missing data and loss to follow-up transparent. Prospective trial within a specified recruitment period. >80% had confirmation of heart failure through echocardiography.Limitations: Representative only of those attending outpatient cardiology services, excluding the acute sector or those patients in primary care with heart failure not under joint care of a cardiologist.
Pakistan [47]	Retrospective	2002–2003	First presentation to Agha Khan University Hospital in Karachi with the diagnosis of new-onset congestive heart failure that met the Boston criteria.Exclusion: LVEF≥40%, prior diagnosis of systolic heart failure dating back 3 mo, underlying disease with expected survival of less than 6 months, known primary valvular heart disease (rheumatic or nonrheumatic), patient died in-hospital, or no follow-up available after discharge.	Clinical diagnosis based on Boston criteria.All patients received echocardiography.	196	Strengths: All patients had echocardiographic assessment.Limitations: Single tertiary referral centre in Karachi may not be generalizable to the broader population. The exclusion of valvular heart disease may impact on the aetiologies ascribed to cases of heart failure. Similarly, the exclusion of those who died in hospital may affect the generalizability of the findings.
Tunisia [70] ∧	Prospective	2008–2009	All outpatients ≥21 y of age with new or previously diagnosed heart failure.Exclusion: patients with acute decompensated heart failure.	Framingham criteria.71% had an echocardiogram.	257	These data come from the I PREFER registry.Strengths: >10 centres. Sites were randomly selected, and all cardiologists within the country considered eligible. Missing data and loss to follow-up transparent. Prospective trial within a specified recruitment period. 71% had confirmation of heart failure through echocardiography.Limitations: Representative only of those attending outpatient cardiology services, excluding the acute sector or those patients in primary care with heart failure not under joint care of a cardiologist.
Yemen [48]	Prospective	2007–2008	First 100 consecutive patients admitted to Ibn Seena Central Hospital, Mukalla, with heart failure. All patients were required to have blood tests, electrocardiogram, echocardiogram, and chest radiogram.Exclusion: all patients who for any reason dropped from follow-up before investigation was completed (died, transferred, discharged)	Framingham criteria.All patients underwent echocardiography.	100	Strengths: Clear diagnostic criteria for underlying aetiologies. All patients had echocardiographic assessment. Referral centre for a large catchment area.Limitations: Single urban tertiary referral centre may not be representative of the broader population of patients with heart failure.

∧ Previously unpublished data.

# S. Rahimzadeh, F. Farzadfar F, and M. Ghaziani, unpublished data.

**Table 4:** Characteristics of Europe region studies and databases included.

Country of Origin	Study Design	Recruitment Period	Selection Criteria	Heart Failure Definition	Cases of Heart Failure	Strengths and Limitations
Romania [51]	Retrospective	2006	459 consecutively admitted patients between January and December 2006 to the cardiology department with a discharge diagnosis of chronic heart failure.	Not specified.	459	Limitations: Single urban general hospital may not be representative of the broader population of patients with heart failure. Diagnostic criteria not clear. Data recorded from hospital files. Unclear how missing data were accounted for.
Romania [50]	Prospective	2008–2009	All consecutive patients hospitalised with a primary diagnosis of acute heart failure syndromes.Exclusion: patients with high-output heart failure.	European Society of Cardiology guidelines.80% of patients had an echocardiogram.	3,224	Strengths: National registry involving 13 sites, increasing the generalizability of its results. A large majority of patients had echocardiographic assessment. Both tertiary academic centres and general hospitals were included, increasing generalizability.Limitations: Unclear how missing data were accounted for, although this issue is acknowledged in their limitations section.
Serbia [61]	Cross-sectional	Unknown	Patients with chronic heart failure were recruited from an outpatient cardiology clinic at the Clinic for Cardiovascular Diseases, Clinical Center Niš.Exclusion: those who had had a worsening of symptoms or changes in treatment in the preceding 2 wk.	European Society of Cardiology and echocardiography.	127	Strengths: All patients underwent echocardiography. Standardised diagnostic criteria.Limitations: Single urban centre may not be representative of the broader population. Unclear method of case ascertainment.
Turkey [49]	Retrospective	1997–1998	Medical records of consecutive patients admitted for congestive heart failure at 16 academic hospitals were selected for review: “The most recent, in average, 50 patients from each centre with sufficient data for CHF [congestive heart failure] in their files were included”.	American Heart Association guidelines.81% had an echocardiogram.	661	Strengths: 16 centres from across the country. A large majority of individuals had echocardiographic assessment.Limitations: Results from academic hospitals may not be generalizable to the broader health system. Method of case ascertainment may lead to selection bias. Unclear how missing data were accounted for.
Turkey [64]	Prospective	1999–2000	A survey was conducted of a random sample of 117 primary care physicians from across Turkey who logged all patients they saw with heart failure.	Not specified.	876	Strengths: Real-world practice taken from a random sample of 117 primary care physicians from across Turkey.Limitations: Diagnosis of heart failure left to the clinicians.
Turkey [63]	Prospective	2005	A sample of 4,650 randomly selected individuals had their height, weight, blood pressure measured as well as an ECG and blood taken for NT-proBNP level. Any of the sample with a cardiac history, abnormal ECG, or NT-proBNP ≥120 pg/ml was further investigated with echocardiography.	Echocardiography.	320	Strengths: Population-based random sample of individuals may provide generalizable information on prevalence of heart failure.

ECG, electrocardiogram.

**Table 5:** Characteristics of South East Asia region studies and databases included.

Country of Origin	Study Design	Recruitment Period	Selection Criteria	Heart Failure Definition	Cases of Heart Failure	Strengths and Limitations
India [69] ∧	Retrospective	2008–2012	Billing codes from hospital used to identify patients with heart failure in Andhra Pradesh.	Not specified.	5,758	Strengths: This study of billing data is from a large sample of over 1.5 million hospitalisations.Limitations: Billing data rely on clinical coding, and consequently there are no standardised diagnostic criteria available.
Indonesia [20]	Prospective	2006	Consecutively hospitalised patients ≥18 y in five hospitals. Patients with heart failure primarily being treated as a co-morbid rather than primary condition.Exclusion: those without an accessible medical record, those without acute decompensated heart failure.	Not specified.	1,687	Indonesian arm of ADHERE-International.Strengths: Five hospitals, improving the potential generalizability of results. Missing data transparently accounted for. Echocardiographic assessment in 37.9% of patients.Limitations: Discharge data with lack of standardisation in the diagnosis of heart failure, which may lead to selection bias.
Thailand [52]	Retrospective	2006–2007	Consecutively hospitalised patients age more than 18 y at 18 cardiovascular centres. Patients with heart failure primarily being treated as a co-morbid rather than primary condition.Exclusion: those without an accessible medical record, patients with cardiogenic shock, and perioperative heart failure.	Not specified.	1,612	Thai arm of ADHERE-International.Strengths: 18 cardiovascular centres from across the country, consequently greater generalizability of the results. 60.4% had echocardiographic assessment.Limitations: Discharge data with lack of standardisation in the diagnosis of heart failure, which may lead to selection bias.

∧ Previously unpublished data.

**Table 6:** Characteristics of Western Pacific region studies and databases included.

Country of Origin	Study Design	Recruitment Period	Selection Criteria	Heart Failure Definition	Cases of Heart Failure	Strengths and Limitations
China [57]	Retrospective	1980–2000	Patients admitted with heart failure to participating hospitals.	Not specified.	1,756	Strengths: Multi-centre study may increase generalizability of results. Long study time period enabling analysis of trends across time.Limitations: Retrospective use of case notes, with consequent lack of standardised diagnostic criteria, may increase selection and reporting bias.
China [58]	Retrospective	1980–2008	Patients admitted to the medical wards during the study period.	Not specified.	2,458	Strengths: Long time period of study allowed the analysis of medication prescription changes over time.Limitations: Urban single-centre study may not be generalizable to broader health service. Retrospective use of case notes without standardised diagnostic criteria for heart failure may increase reporting and selection bias.
China [56]	Retrospective	1995–2004	Patients admitted with heart failure.	Not specified.	259	Limitations: Diagnostic criteria not standardised, leading to potential for reporting and selection bias.
China [53]	Retrospective	1995–2009	Patients admitted with heart failure to three university hospitals.	American Heart Association 2005 guidelines.	1,119	Strengths: Multi-centre cohort study.Limitations: Academic centres may not reflect the broader health service.
China [55]	Retrospective	2007	Patients admitted with heart failure.	Framingham criteria.	478	Limitations: Rural single-centre study that may not be generalizable to the broader health service. Retrospective analysis of case notes open to reporting bias.
China [54]	Retrospective	2008–2009	Patients admitted with heart failure.	European Society of Cardiology 2005 guidelines	206	Limitations: Urban single-centre study that may not be generalizable to the broader health service.
China [60]	Prospective	Unknown (published 2012)	Individuals admitted to the People's Liberation Army General Hospital, Beijing, over the age of 60 y with a diagnosis of chronic heart failure.Exclusion: those with severe aortic stenosis, anticipated cardiac transplantation, or a left ventricular assist device.	European Society of Cardiology 2008 guidelines.	327	Limitations: Single-centre army general hospital in the capital with a high proportion of male patients (78% of this cohort) may not be broadly generalizable.
China [62]	Prospective	Unknown (published 2009)	Cluster randomised sample of adults in primary care facilities with congestive heart failure in six counties of Liaoning Province.	Framingham criteria.	529	Strengths: Cluster randomisation of primary care facilities, reducing potential for bias. Representation from six counties of Liaoning Province may improve regional generalizability of the results.Limitations: Uncertain diagnostic criteria and patient pool.
Malaysia [68] ∧	Retrospective	2007–2008	Consecutive individuals with a principal discharge diagnosis of heart failure, using the relevant International Classification of Disease-9 codes. Patients with heart failure primarily being treated as a co-morbid rather than primary condition.Exclusion: patients <18 y, those without an accessible medical record, those without acute decompensated heart failure.	Not specified.	907	Malaysian arm of ADHERE-International.Strengths: Multi-centre trial, improving the potential generalizability of results. All questions on the electronic case report were required to be completed, eliminating reporting bias due to missing data.Limitations: Discharge data with lack of standardisation in the diagnosis of heart failure, which may lead to selection bias.
Philippines [68] ∧	Retrospective	2006–2007	All consecutive individuals admitted with an International Classification of Disease-9 code for heart failure. Patients with heart failure primarily being treated as a co-morbid rather than primary condition.Exclusion: Patients <18 y, those without an accessible medical record, and those without acute decompensated heart failure.	Not specified.	261	Philippines arm of ADHERE-International.Strengths: Multi-centre trial, improving the potential generalizability of results. All questions on the electronic case report were required to be completed, eliminating reporting bias due to missing data.Limitations: Discharge data with lack of standardisation in the diagnosis of heart failure, which may lead to selection bias.

∧ Previously unpublished data.

### Statistical Analysis

Study-specific data on percentages are presented as forest plots with exact binomial 95% confidence intervals (CIs). These percentages were pooled, by World Health Organization region (Table 7) and across regions, using the random effects method of DerSimonian and Laird [17]. Heterogeneity between studies was quantified by the *I* ^2^ statistic and tested using Cochran's *Q* test. Means were rarely reported with an estimate of variability, and, consequently, we weighted individual means by study size in pooled analyses, and present the pooled mean and the range of means.

**Table 7:** Included countries grouped by World Health Organization region.

Africa	Americas	Eastern Mediterranean	Europe	South East Asia	Western Pacific
Algeria	Argentina	Egypt	Romania	India	China
Cameroon	Brazil	Iran	Turkey	Indonesia	Malaysia
DRC	Chile	Lebanon	Serbia	Thailand	Philippines
Ethiopia	Colombia	Pakistan			
Ghana	Mexico	Tunisia			
Kenya		Yemen			
Mozambique					
Nigeria					
Senegal					
South Africa					
Uganda					

DRC, Democratic Republic of the Congo.

Patients presenting acutely to hospitals may differ in many respects from those that are seen in clinics for chronic management. When pooling the data we therefore indicate the setting of each study in all forest plots. Studies from community primary care or outpatient clinics were designated as non-acute, and studies from inpatient populations, acute. Studies reporting both inpatient and outpatient data were included in the non-acute category. Additional subgroup analyses were performed by level of country income and by study time period. For income level analyses, studies were divided into low-income, low-middle-income, and upper-middle-income groups according to World Bank [15] country classification at the final year of the recruitment period of the study. The relationship between a study's mean age at admission for heart failure and the human development index (HDI) [18] for the country involved was estimated with linear regression analysis; the HDI was taken for the closest year to the final year of patient recruitment for the studies representing each country. The HDI is a composite measure of development produced by the United Nations Development Programme that incorporates life expectancy, education, and gross national income per capita [18]. Random effects meta-regression was performed to investigate study year as an explanation for the between-study heterogeneity in causes of heart failure, management, and in-hospital mortality. Corresponding bubble plots were drawn, with the size of each bubble inversely proportional to the estimated variance in the respective study.

Statistical analyses were done using R version 3.0.2 and Stata version 11.2.

## Results

### Geographic Distribution and Study Description

**Figure 2:**
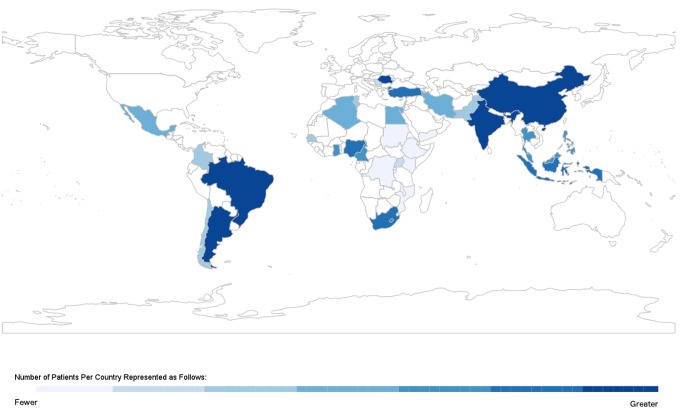
Geographic distribution of studies on heart failure in lowand middle-income countries.

Overall, 49 published studies [19]–[67] and four unpublished datasets ([68]–[70]; S. Rahimzadeh, F. Farzadfar F, M. Ghaziani, unpublished data) were included; their geographical distribution is presented in Figure 2, and key study characteristics, divided by WHO region, are summarised in Tables 1–6. We obtained unpublished country datasets from the Acute Decompensated Heart Failure Registry (ADHERE)–International [68] regarding Malaysia and the Philippines, as well as the Identification of Patients with Heart Failure and Preserved Systolic Function (I PREFER) registry [70] including Iran, Lebanon, Egypt, Tunisia, Algeria, Chile, Colombia, and Mexico. Additional unpublished data were contributed from Iran (S. Rahimzadeh, F. Farzadfar F, and M. Ghaziani, unpublished data) and India [69].

Most studies were based in a single hospital, although 21 datasets documented multi-centre studies in Algeria [70], Argentina [21],[43],[44], Brazil [40],[45], Chile [41],[70], Colombia [70], China [55],[57],[62], Egypt [70], Indonesia [20], India [69], Iran (S. Rahimzadeh, F. Farzadfar F, and M. Ghaziani, unpublished data; [70]), Lebanon [70], Thailand [52], Malaysia [68], Mexico [70], Philippines [68], Romania [50], Tunisia [70], Turkey [49],[63],[64], and a further nine countries in sub-Saharan Africa [35]. Four studies involved both inpatient and outpatient data [22],[24],[26],[36], six studies referred solely to patients seen at outpatient clinics [27],[39],[61],[66],[67],[70], three studies described heart failure in primary care settings [62]–[64], and the remainder reported solely on inpatient populations. One study was a population-based assessment of the prevalence of heart failure in Turkey [63].

### Case Identification and Ascertainment

The studies together included 237,908 episodes of heart failure hospitalisation. The median number of cases across all studies was 386 (range: 100–194,098). Diagnosis of heart failure was established according to the Framingham criteria [71] in 12 studies [21],[22],[24],[28]–[31],[42],[48],[55],[62],[70]. European Society of Cardiology guidelines were used in eight studies [25]–[27],[41],[50],[54],[60],[61], the Boston criteria [72] in three studies [39],[40],[47], and the American Heart Association guidelines in two studies [49],[53], and the diagnosis was left to the investigator's or examining physician's discretion in 26 studies ([19],[20],[23],[32]–[38],[43]–[46],[51],[52],[56]–[58],[64],[65],[67]–[69]; S. Rahimzadeh, F. Farzadfar F, and M. Ghaziani, unpublished data). One study diagnosed all cases of heart failure solely using echocardiography [59]. Information on the use of additional investigative tools, including echocardiography, chest radiography, and electrocardiography, was provided in 28 studies [19]–[21],[24]–[26],[28],[31],[35],[39],[41]–[43],[46],[48]–[50],[51],[54],[56],[57],[59],[61],[63],[64],[66],[70],[73]. Of these, 14 studies performed echocardiography on all patients [19],[24]–[26],[28],[35],[46],[48],[53],[54],[59],[61],[63],[66]. The mean left ventricular ejection fraction (LVEF) was documented in 18 studies, reporting data from Algeria [70], Egypt [70], Tunisia [70], Cameroon [24], Ethiopia [35], Sudan [35], Mozambique [35], Kenya [35], Uganda [35], Senegal [35], South Africa [25], Nigeria [26],[28], Brazil [19],[39],[42],[46],[66], Chile [41],[70], Colombia [70], Mexico [70], Romania [50], Serbia [61], Turkey [49], Iran [70], Lebanon [70], Indonesia [20], Thailand [52], and China [53]. Across all studies, mean LVEF was 40% (range: 27%–57%) (Table 8). Hospitalised patients had a mean LVEF of 38% (27%–57%), with a corresponding figure of 48% (29%–55%) in non-acute settings.

**Table 8:** Characteristics of patients, by region.

Characteristic	Region						
	Africa	Americas	Eastern Mediterranean	Europe	South East Asia	Western Pacific	All
**Age**							
Mean age (range), in years*	52 (42–64)	70 (53–77)	63 (57–69)	67 (61–73)	54 (50–64)	67 (53–74)	63 (42–77)
Number of studies	14	14	4	5	3	7	45
**Male**							
Percent male (95% CI)	51% (43%–59%)	58% (54%–63%)	65% (61%–70%)	61% (48%–73%)	60% (51%–70%)	58% (50%–65%)	58% (54%–62%)
*I* ^2^ (95% CI)	99% (98%–99%), *p*<0.001	98% (98%–99%), *p*<0.001	61% (6%–84%), *p*<0.0239	99% (98%–99%), *p*<0.001	99% (98%–99%), *p*<0.001	98% (97%–99%), *p*<0.001	100% (100%–100%), *p*<0.001
Number of studies	13	16	3	6	3	9	48
**LVEF**							
Mean (range) LVEF, in percent*	42% (29%–49%)	41% (27%–43%)	50% (34%–55%)	38% (38%–40%)	33% (—)	42% (38%–57%)	40% (27%–57%)
Number of studies	6	7	1	3	1	2	17
**Length of stay**							
Mean (range) number of days*	11 (9–13)	10 (5–25)	5 (—)	—	3 (—)	23 (13–35)	10 (5–35)
Number of studies	3	6	1	—	1	3	14

*Weighted by study size.

### Demographic Characteristics

**Figure 3:**
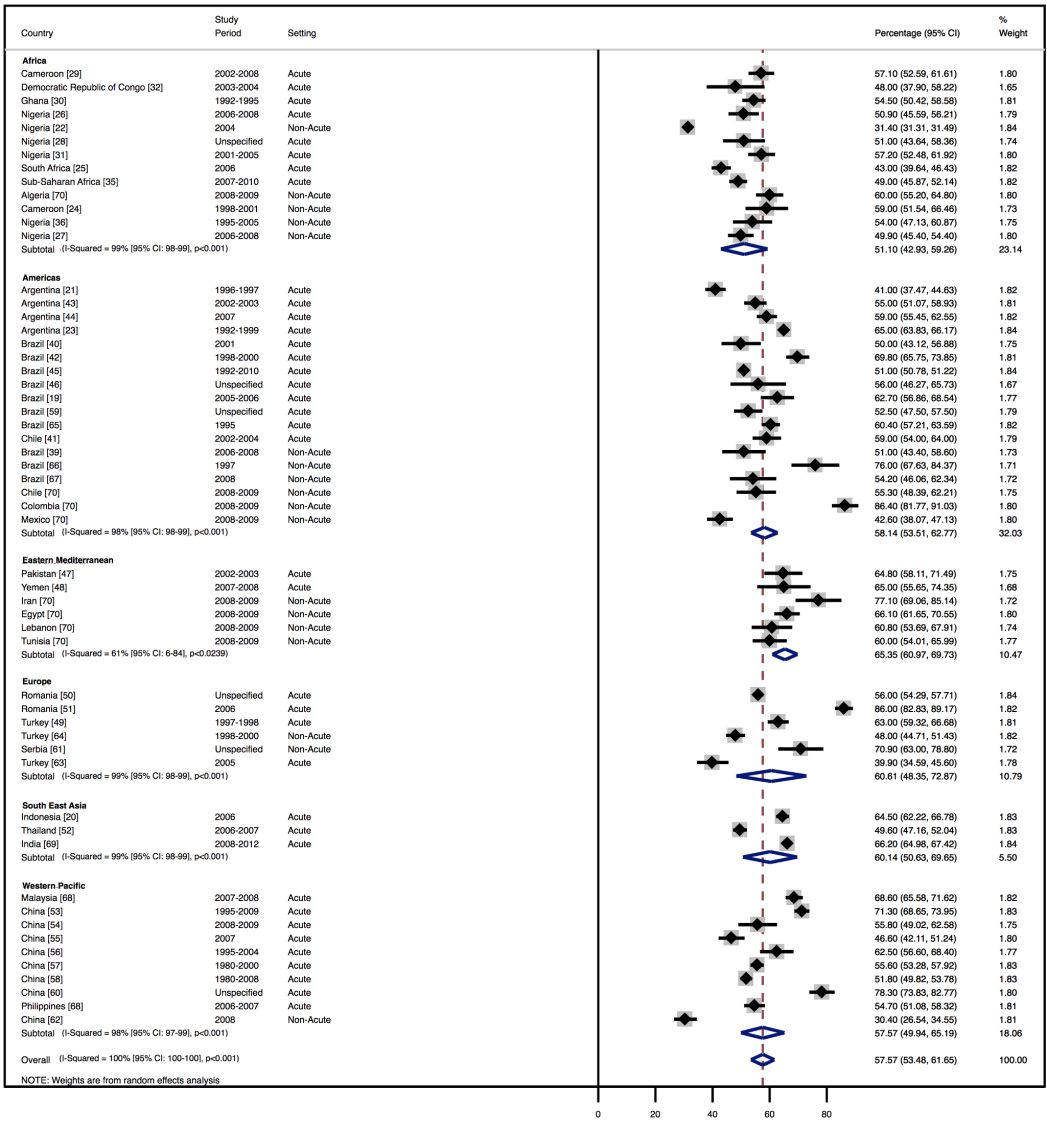
Male patients by region.

**Figure 4:**
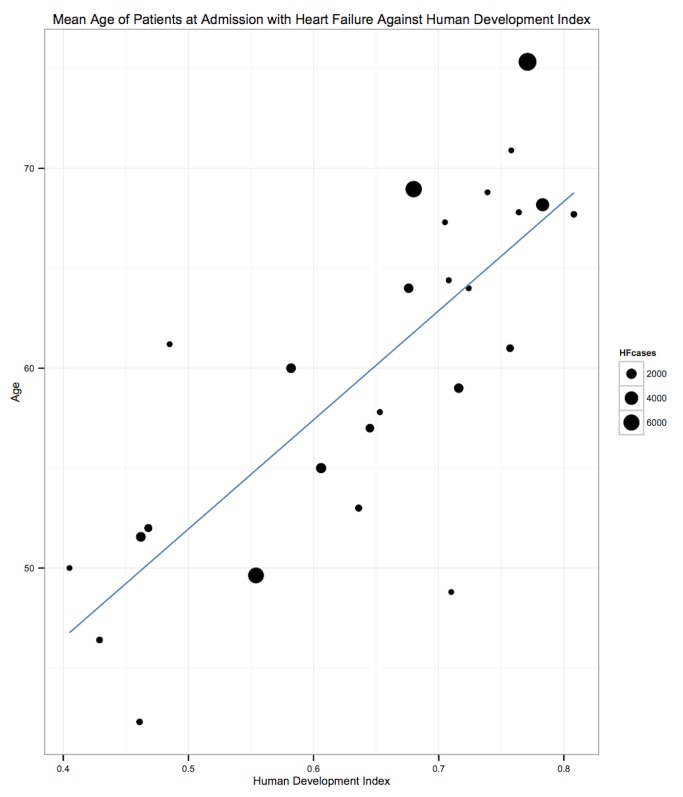
Correlation of age and human development index, by country. *r* = 0.71, *p*<0.001. The HDI is a measure produced by the United Nations Development Programme that incorporates gross national income per capita, life expectancy, and time spent in education. It serves as a single statistic that provides a comparable measure of development across nations. HF, heart failure.

The demographic characteristics of patients and outcomes by region are shown in Table 8. The corresponding data by country are shown in Table 9. Men made up 58% (95% CI: 54%–62%) of study participants (Figure 3). The mean age of patients for each region ranged from just over 52 y (range: 42–64) in Africa to 70 y (range: 53–77) in the Americas, and when combined across all regions was 63 y (range: 42–77). The mean age of patients on admission rose with the country income level. In low-income countries, the corresponding figure was 50 y (range: 42–58), rising to 60 y (range: 50–74) in low-middle-income countries, and reaching 70 y (range: 54–77) in upper-middle-income countries. Mean age also correlated with the HDI across countries (*r* = 0.71, *p*<0.001) (Figure 4).

**Table 9:** Characteristics of patients, by country.

Region and Country	Recruitment Period	Heart Failure Cases	Mean Age (Years)	Male (Percent)	Mean Length of Stay (Days)	Mean LVEF (Percent)
**Africa**						
Algeria [70] ∧	2008–2009	400	64	60%	—	49%
Cameroon [24]	1998–2001	167	57	59%	—	23%
Cameroon [29]	2002–2008	462	43	57%	13	
Democratic Republic of the Congo [32]	2003–2004	100	57	48%	—	
Ghana [30]	1992–1995	572	42	55%	—	
Nigeria [26]	2006–2008	340	51	51%	—	42%
Nigeria [27]	2006–2010	475	49	50%	—	
Nigeria [22]	2004	102	45	31%	—	
Nigeria [28]	Unspecified	177	52	51%	—	45%
Nigeria [31]	2001–2005	423	54	57%	—	
Nigeria [36]	1995–2005	202	56	54%	—	
Senegal [38]	2001	170	50	—	11	
South Africa [25]	2006	844	55	43%	—	45%
Sub-Saharan Africa [35]	2007–2010	1,006	52	49%	9	40%
**Americas**						
Argentina [21]	1996–1997	751	66	41%	—	
Argentina [43]	2002–2003	615	70	55%	—	
Argentina [44]	2007	736	—	59%	—	
Argentina [23]	1992–1999	6,368	77	65%∧	5	
Brazil [39]	2006–2008	166	61	51%	—	49%
Brazil [40]	2001	203	67	50%	8*	
Brazil [42]	1998–2000	494	58	70%	—	34%
Brazil [45]	1992–2010	194,098	—	51%	10	
Brazil [46]	Unspecified	100	59	56%	9	46%
Brazil [19]	2005–2006	263	60	63%	25	27%
Brazil [59]	Unspecified	383	54	53%	—	
Brazil [66]	1997	100	57	76%	—	43%
Brazil [67]	2008	144	61	54%	—	
Brazil [65]	1995	903	53	60%	—	
Chile [41]	2002–2004	372	69	59%	11	35%
Chile [70] ∧	2008–2009	199	65	55%	—	42%
Colombia [70] ∧	2008–2009	211	70	86%	—	46%
Mexico [70] ∧	2008–2009	458	68	43%	—	54%
**Eastern Mediterranean**						
Egypt [70] ∧	2008–2009	434	58	66%	—	55%
Iran# ^,^ ∧	1998–2012	277	67	—	5	
Iran [70] ∧	2008–2009	105	57	77%	—	34%
Lebanon [70] ∧	2008–2009	181	69	61%	—	43%
Pakistan [47]	2002–2003	196	61	65%	—	
Tunisia [70] ∧	2008–2009	257	67	51%	—	53%
Yemen [48]	2007–2008	100	58	65%	—	
**Europe**						
Romania [50]	2008–2009	3,224	69	56%	—	38%
Romania [51]	2006	459	61	86%	—	
Serbia [61]	Unspecified	127	71	73%	—	40%
Turkey [49]	1997–1998	661	61	64%	—	38%
Turkey [63]	2005	320	—	40%	—	
Turkey [64]	1998–2000	876	64	48%	—	
**South East Asia**						
India [69] ∧	2008–2012	5,758	50	66%	3	
Indonesia [20]	2006	1,687	60	65%	—	33%
Thailand [52]	2006–2007	1,612	64	50%	—	
**Western Pacific**						
China [53]	1995–2009	1,119	65	71%	—	38%
China [54]	2008–2009	206	74	56%	—	
China [55]	2007	478	69	47%	—	
China [56]	1995–2004	259	70	63%	29	
China [57]	1980–2000	1,756	68	56%	35	
China [58]	1980–2008	2,458	71	52%	13	
China [60]	Unspecified	327	—	78%	—	57%
China [62]	2008	529	—	30%	—	
Malaysia [68] ∧	2007–2008	907	61	69%	—	
Philippines [68] ∧	2006–2007	725	53	55%	—	

∧ Previously unpublished dataset.

*Contributed by author.

# S. Rahimzadeh, F. Farzadfar F, and M. Ghaziani, unpublished data.

### Causes of Heart Failure

**Figure 5:**
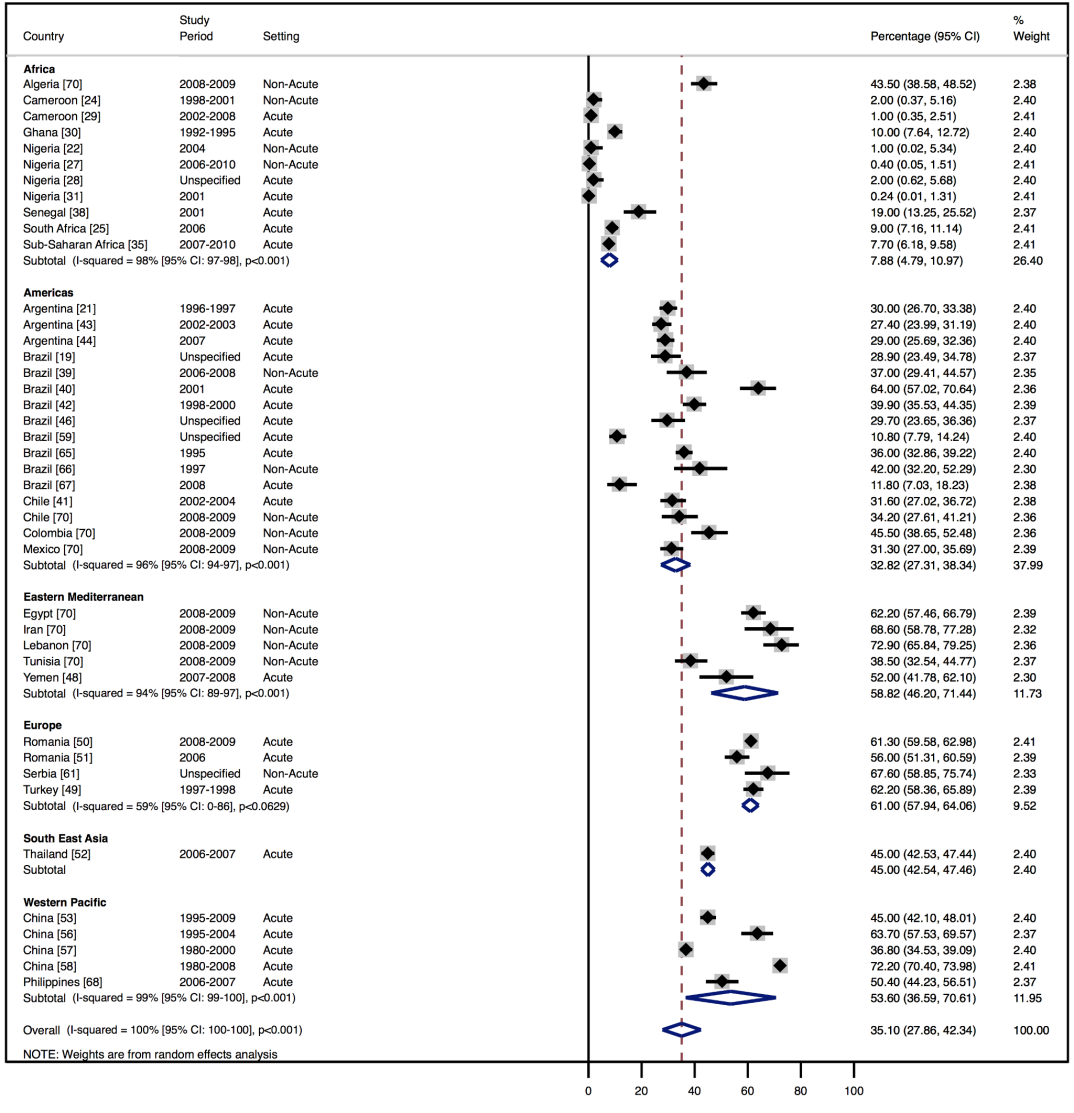
Aetiology of heart failure: ischaemic heart disease by region. Percentage of heart failure cases with a documented cause of IHD.

**Figure 6:**
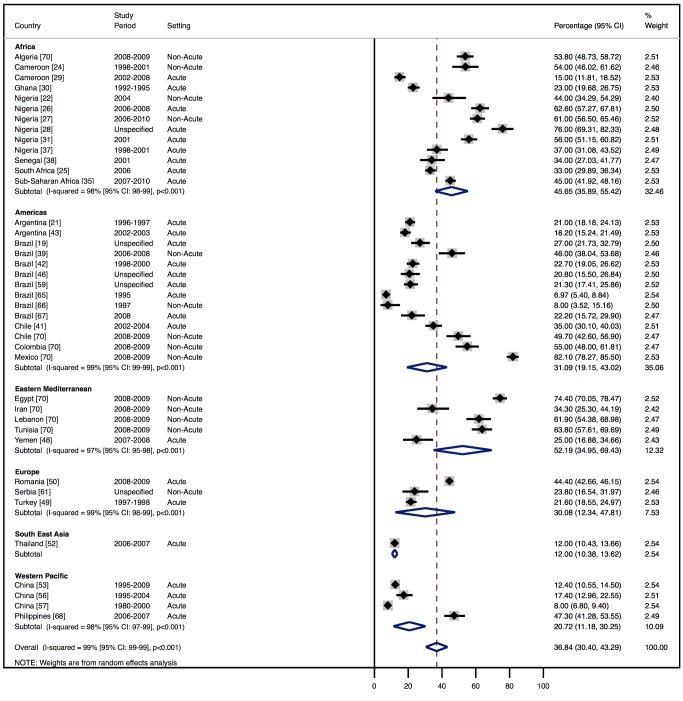
Aetiology of heart failure: hypertension by region. Percentage of heart failure cases with a documented cause of hypertension.

**Figure 7:**
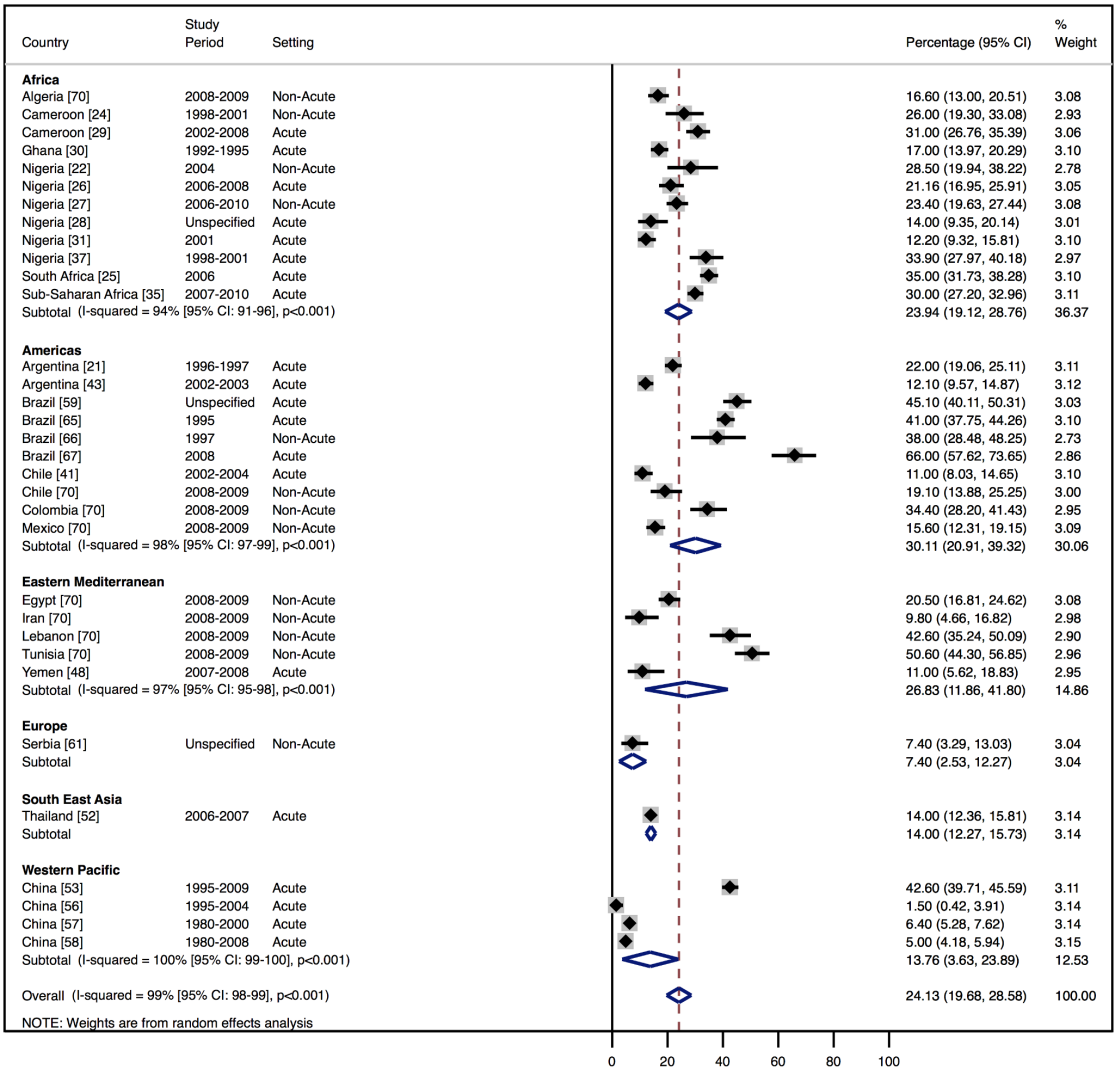
Aetiology of heart failure: cardiomyopathies by region. Percentage of heart failure cases with a documented cause of cardiomyopathy.

**Figure 8:**
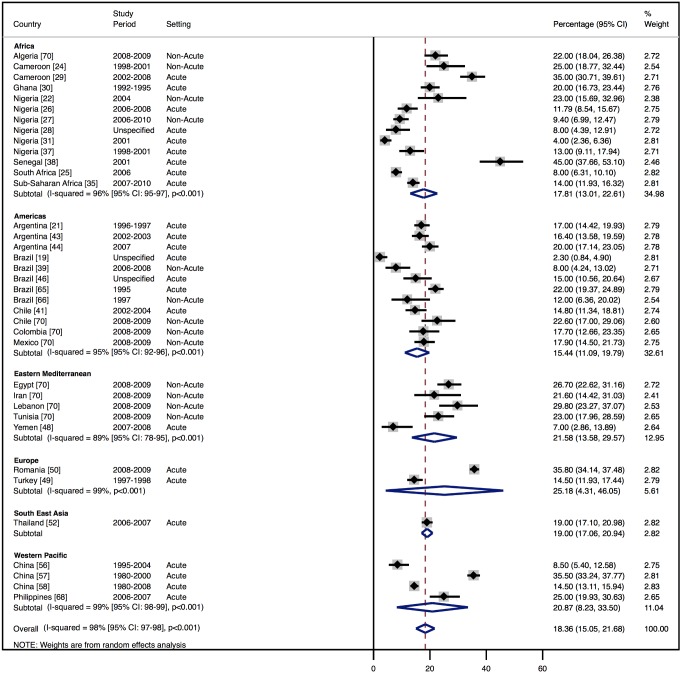
Aetiology of heart failure: valvular heart disease by region. Percentage of heart failure cases with a documented cause of valvular heart disease.

Although most studies made a clear distinction between aetiologies and co-morbidities, the categories reported were highly variable, and multiple causes were often attributed to individual cases of heart failure. Across all LMICs, non-communicable diseases, and in particular ischaemic heart disease (IHD) and hypertension, are the leading causes of heart failure (Table 10). However, there is heterogeneity between the regions. IHD is the most commonly reported cause of heart failure in all regions except Africa and the Americas (Figures 5 and 6). In the Americas hypertension and IHD are responsible for a similar percentage of documented cases, at 31% (95% CI: 19%–43%, *I* ^2^ 99%, *p* for heterogeneity <0.001) and 33% (95% CI: 27%–38%, *I* ^2^ 96%, *p*<0.001), respectively. In Africa, 8% (95% CI: 5%–11%, *I* ^2^ 98%, *p*<0.001) of heart failure is due to IHD, with hypertension the dominant cause, responsible for 46% (95% CI: 36%–55%, *I* ^2^ 98%, *p*<0.001) of cases. Cardiomyopathies cause 24% (95% CI: 20%–29%, *I* ^2^ 99%, *p*<0.001) of heart failure cases across LMICs taken together (Figure 7). Idiopathic, hypertrophic, and restrictive cardiomyopathies are reported across all countries; however, other specific types of cardiomyopathies showed substantial regional variation. Peri-partum and HIV-associated cardiomyopathies were reported only in Africa. By contrast, Chagas cardiomyopathy remains a Latin American phenomenon [21],[43]. Valvular heart disease is responsible for 18% (95% CI: 15%–22%, *I* ^2^ 98%, *p*<0.001) of cases of heart failure across LMICs (Figure 8).

**Table 10:** Reported causes of heart failure, by region.

Cause	Region						
	Africa	Americas	Eastern Mediterranean	Europe	South East Asia	Western Pacific	All
**Hypertension**							
Percent (95% CI)	46% (36%–55%)	31% (19%–43%)	52% (35%–69%)	30% (12%–48%)	12% (10%–14%)	21% (11%–30%)	37% (30%–43%)
*I* ^2^ (95% CI)	98% (98%–99%), *p*<0.001	99% (99%–99%), *p*<0.001	97% (95%–98%), *p*<0.001	99% (98%–99%), *p*<0.001	—	98% (97%–99%), *p*<0.000	99% (99%–99%), *p*<0.001
Number of studies	13	12	2	3	1	4	33
**IHD**							
Percent (95% CI)	8% (5%–11%)	33% (27%–38%)	59% (46%–71%)	61% (58%–64%)	45% (43%–48%)	54% (37%–71%)	35% (28%–42%)
*I* ^2^ (95% CI)	98% (97%–98%), *p*<0.001	96% (94%–97%), *p*<0.001	94% (89%–97%), *p*<0.001	59% (0%–86%), *p*<0.063	—	99% (99%–100%), *p*<0.001	100% (100%–100%), *p*<0.001
Number of studies	11	14	2	4	1	5	35
**Valvulopathy**							
Percent (95% CI)	18% (13%–23%)	15% (11%–20%)	22% (14%–30%)	25% (4%–46%)	19% (17%–21%)	21% (8%–34%)	18% (15%–22%)
*I* ^2^ (95% CI)	96% (95%–97%), *p*<0.001	95% (92%–96%), *p*<0.001	89% (78%–95%), *p*<0.001	99% (—), *p*<0.001	—	99% (98%–99%), *p*<0.001	98% (97%–98%), *p*<0.001
Number of studies	13	9	2	2	1	4	29
**Cardiomyopathy**							
Percent (95% CI)	24% (19%–29%)	30% (21%–39%)	27% (12%–42%)	7% (3%–12%)	14% (12%–16%)	14% (4%–24%)	24% (20%–29%)
*I* ^2^ (95% CI)	94% (91%–96%), *p*<0.001	98% (97%–99%), *p*<0.001	97% (95%–98%), *p*<0.001	—	—	99% (99%–100%), *p*<0.001	99% (98%–99%), *p*<0.001
Number of studies	12	7	2	1	1	4	26

### Management of Heart Failure

**Figure 9:**
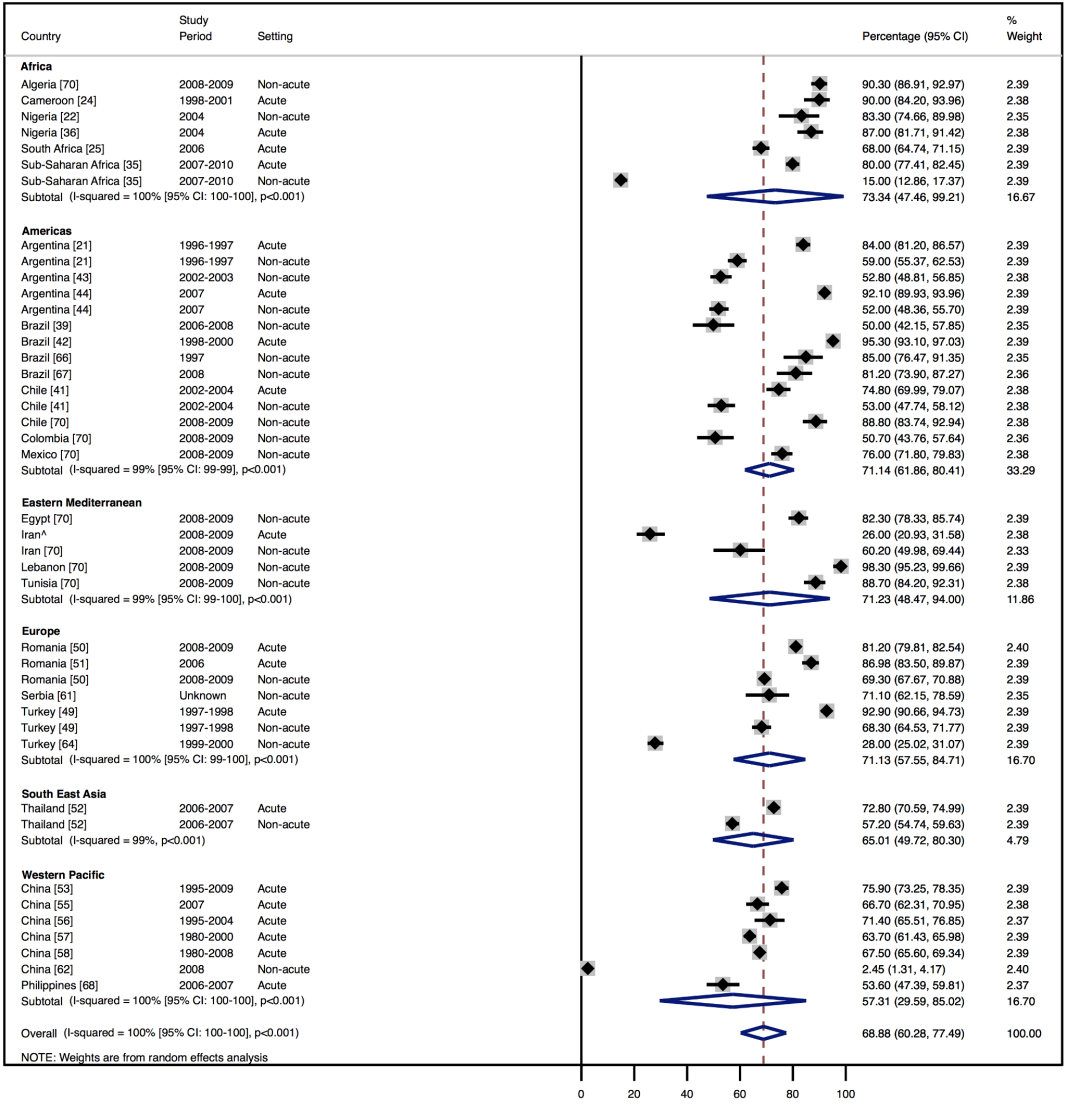
Diuretic use by region. Loop and/or thiazide diuretics. ∧Rahimzadeh S, Farzadfar F, Ghaziani M (2013) Iranian hospital data project (unpublished data).

**Figure 10:**
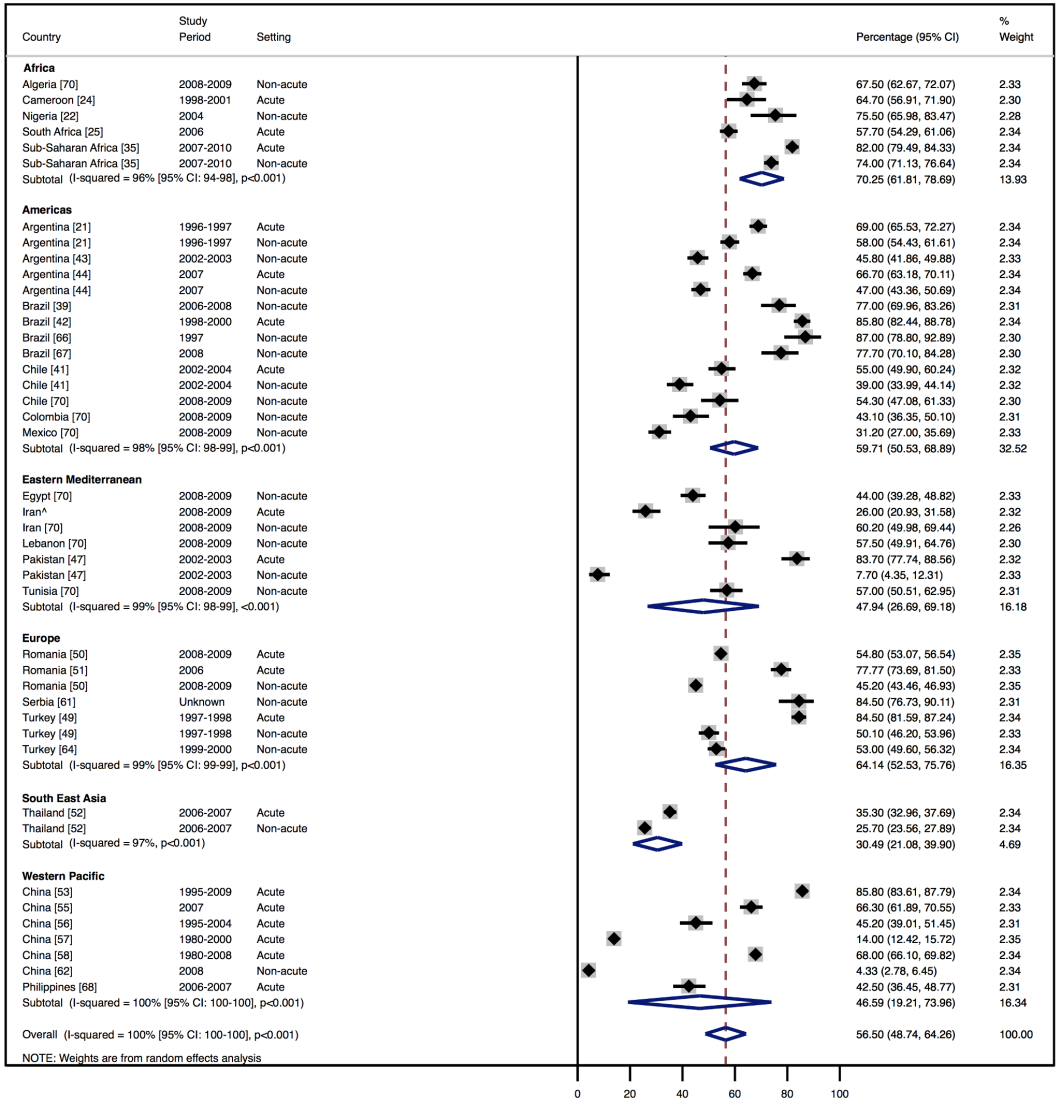
Angiotensin-converting enzyme inhibitor use by region. ∧Rahimzadeh S, Farzadfar F, Ghaziani M (2013) Iranian hospital data project (unpublished data).

**Figure 11:**
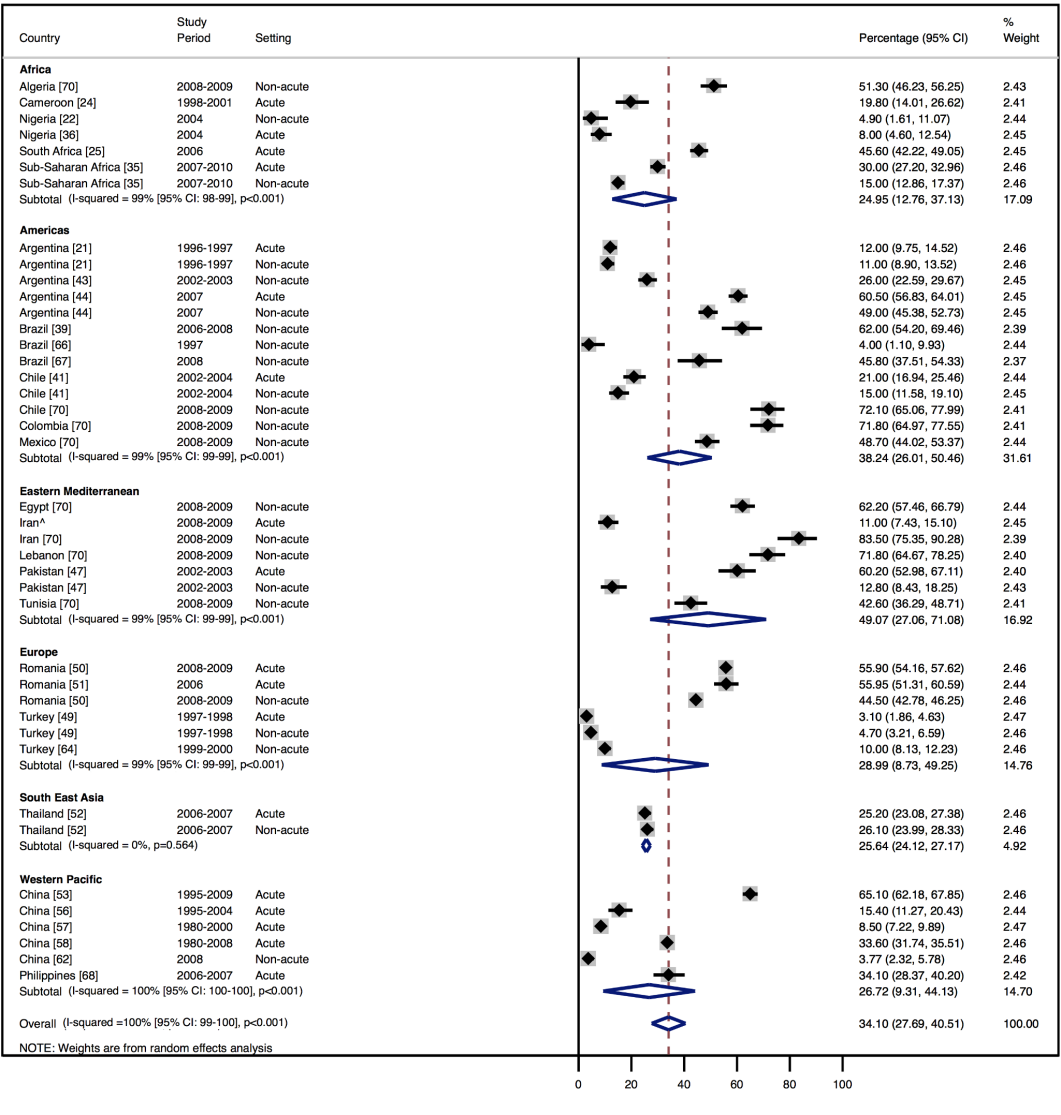
Beta-blocker use by region. ∧Rahimzadeh S, Farzadfar F, Ghaziani M (2013) Iranian hospital data project (unpublished data).

**Figure 12:**
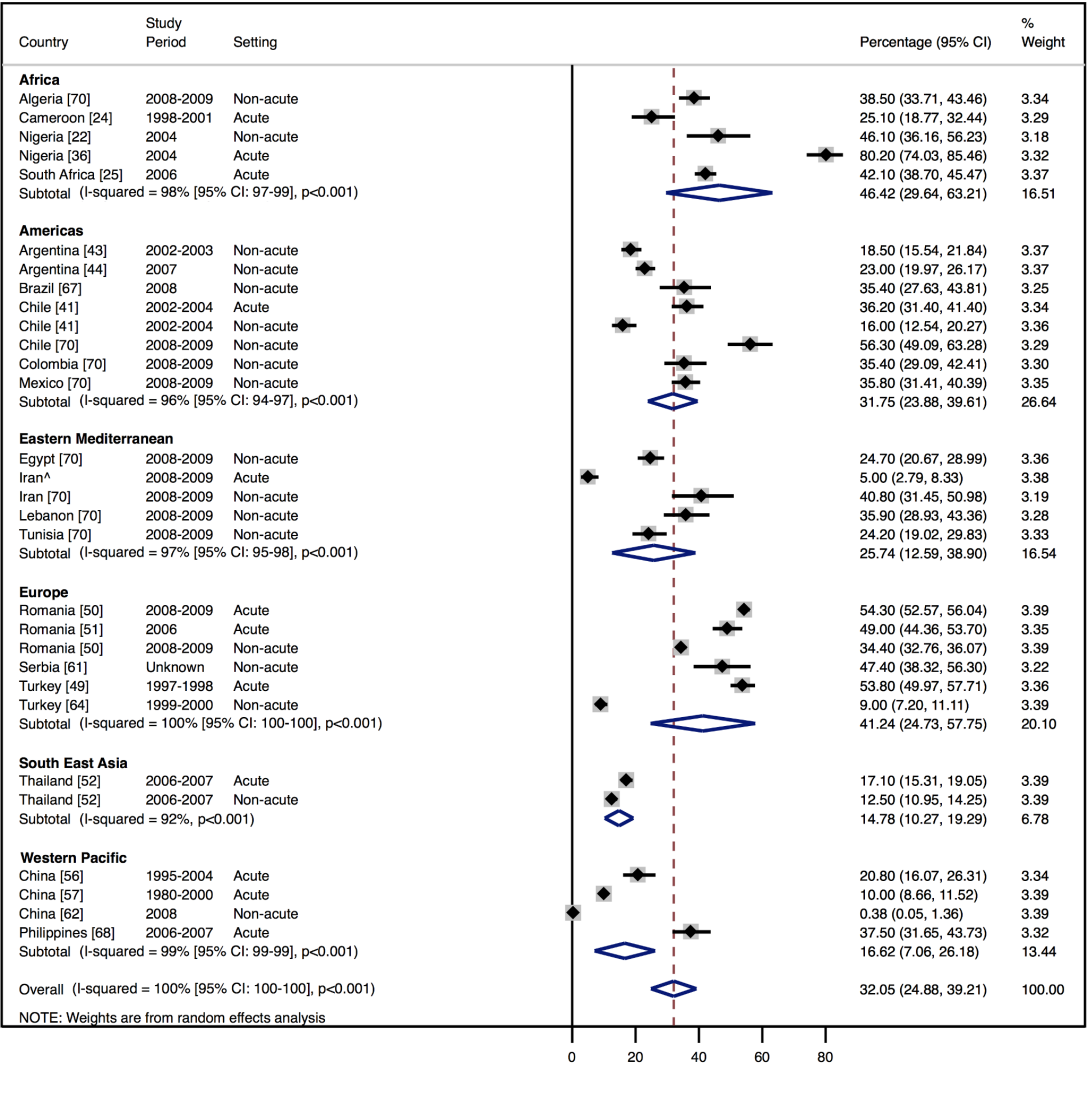
Mineralocorticoid receptor antagonist use by region. ∧Rahimzadeh S, Farzadfar F, Ghaziani M (2013) Iranian hospital data project (unpublished data).

Amongst all studies, the management of heart failure varies considerably between regions and within regions, as well as between studies from the same country (Table 11). The most commonly prescribed treatments are loop and/or thiazide diuretics, prescribed for 69% (95% CI: 60%–78%, *I* ^2^ 100%, *p*<0.001) of individuals in LMICs worldwide (Figure 9). Angiotensin-converting enzyme inhibitors (ACEIs) are used in 57% (95% CI: 49%–64%, *I* ^2^ 100%, *p*<0.001) of cases, beta-blockers in 34% (95% CI: 28%–41%, *I* ^2^ 100%, *p*<0.001), and mineralocorticoid receptor antagonists in 32% (95% CI: 25%–39%, *I* ^2^ 100%, *p*<0.001) (Figures 10–12).

**Table 11:** Reported management of heart failure, by region.

Management	Region						
	Africa	Americas	Eastern Mediterranean	Europe	South East Asia	Western Pacific	All
**ACEIs**							
Percent (95% CI)	70% (62%–79%)	60% (51%–69%)	48% (27%–69%)	64% (53%–76%)	31% (21%–40%)	47% (19%–74%)	57% (49%–64%)
*I* ^2^ (95% CI)	96% (94%–98%), *p*<0.001	98% (98%–99%), *p*<0.001	99% (99%–99%), *p*<0.001	99% (99%–99%), *p*<0.001	97% (—), *p*<0.001	100% (100%–100%), *p*<0.001	100% (100%–100%), *p*<0.001
Number of studies	6	9	3	5	1	7	29
**Beta-blockers**							
Percent (95% CI)	25% (13%–37%)	38% (26%–51%)	49% (27%–71%)	29% (9%–49%)	26% (24%–27%)	27% (9%–44%)	34% (28%–41%)
*I* ^2^ (95% CI)	99% (98%–99%), *p*<0.001	99% (99%–99%), *p*<0.001	99% (99%–99%), *p*<0.001	100% (100%–100%), *p*<0.001	0% (—), *p*<0.564	100% (100%–100%), *p*<0.001	100% (99%–100%), *p*<0.001
Number of studies	7	8	3	4	1	5	26
**Diuretics**							
Percent (95% CI)	73% (48%–99%)	71% (62%–80%)	71% (49%–94%)	71% (58%–85%)	65% (50%–80%)	57% (30%–85%)	69% (60%–78%)
*I* ^2^ (95% CI)	100% (100%–100%), *p*<0.001	99% (99%–99%), *p*<0.001	99% (99%–100%), *p*<0.001	100% (99%–100%), *p*<0.001	99% (—), *p*<0.001	100% (100%–100%), *p*<0.001	100% (100%–100%), *p*<0.001
Number of studies	6	9	2	5	1	6	27
**Mineralocorticoid receptor antagonists**							
Percent (95% CI)	46% (30%–63%)	32% (24%–40%)	26% (13%–39%)	41% (25%–58%)	15% (10%–19%)	17% (7%–26%)	32% (25%–39%)
*I* ^2^ (95% CI)	98% (97%–99%), *p*<0.001	96% (94%–97%), *p*<0.001	97% (95%–98%), *p*<0.001	100% (100%–100%), *p*<0.001	92% (—), *p*<0.001	99% (99%–99%), *p*<0.001	100% (100%–100%), *p*<0.001
Number of studies	5	5	2	5	1	4	20

### Outcomes

Across LMICs, patients admitted with heart failure remained in hospital for a mean of 10 d (Table 8). The mean hospital stay ranged from 3 d in India to 23 d amongst studies from China. Wide differences were observed amongst Argentinian and Brazilian studies. In Argentina, length of stay varied between studies from 5 d to 25 d, with an overall mean of 7 d. In Brazil, the range was between 9 and 25 d, with an overall mean of 10 d (see Table 9 for individual study data).

**Figure 13:**
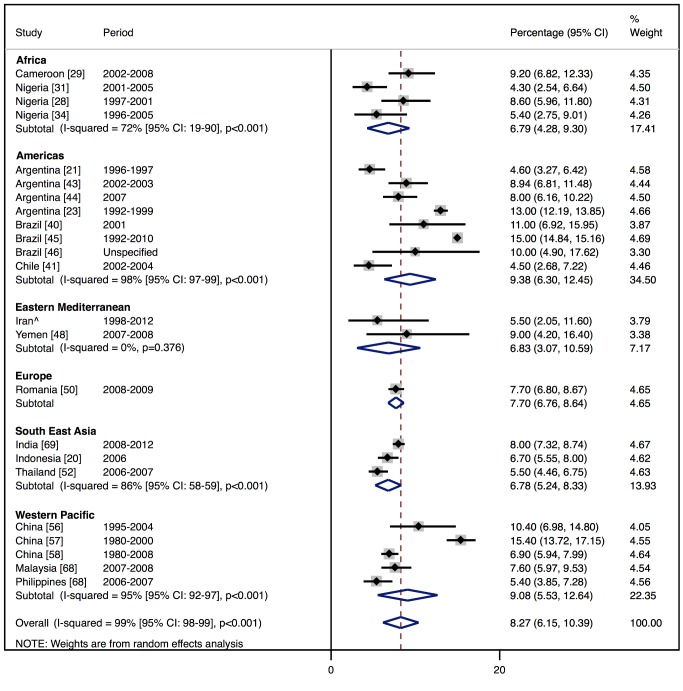
In-hospital mortality rates by region. ∧Rahimzadeh S, Farzadfar F, Ghaziani M (2013) Iranian hospital data project (unpublished data).

In-hospital mortality was 8% (95% CI: 6%–10%, *I* ^2^ 99%, *p*<0.001) (Figure 13) across the 23 studies that reported this measure ([20],[21],[23],[29],[31],[33]–[35],[40],[41],[43]–[46],[48],[50],[52],[56]–[58],[68]–[69]; S. Rahimzadeh, F. Farzadfar F, and M. Ghaziani, unpublished data), with no significant association with the country-level length of stay. Four studies reported longer-term outcome data, showing comparable mortality rates post-discharge: THESUS-HF, a multi-centre study of heart failure across nine countries of sub-Saharan Africa, found that mortality from heart failure was 4.2% in hospital and 17.8% at 6 mo after hospital discharge [35]. In Brazil, Barretto and colleagues reported a mortality rate of 8.8% in hospital and 25.8% at 1 y [19], whilst in Pakistan, after almost a year of follow-up, a similar mortality rate of 27.5% was recorded [47]. Of the Brazilian cohort studied by de Campos Lopes and colleagues, 44% were alive at 21 mo of follow-up [42].

Data relating to heart failure as a proportion of total hospital admissions were available for five countries. Across these countries, heart failure accounted for 2.2% (range: 0.3%–7.7%) of total admissions. Brazil was the only LMIC with nationwide registry data compiled for all patients with heart failure treated by its public health system [74]. Here, heart failure was responsible for 2.2% of total hospitalisations across the country [74]. In India, heart failure accounted for only 0.37% of cases from a sample of 1,551,410 hospitalisations, as derived from billing data in Andhra Pradesh [69]. Out of a representative sample of 38,926 hospital admissions in Iran, 0.3% were identified as having heart failure as the primary cause of admission (S. Rahimzadeh, F. Farzadfar F, and M. Ghaziani, unpublished data). By contrast, 5.8% of total hospital admissions in Cameroon were due to heart failure [24],[29], and 7.7% in Argentina [23].

In sub-Saharan Africa, the total number of cardiovascular admissions was reported, rather than total hospital admissions. In Nigeria, heart failure accounted for 31% of cardiovascular cases presenting to hospital [27], with corresponding figures of 38% in Senegal [38] and 47% in Soweto, South Africa [75].

Population-level data regarding the prevalence of heart failure were available in only one study, from Turkey [63]. Here an absolute prevalence of 2.9% for heart failure was found across the sample [63].

### Effect of Time on Heterogeneity of Outcomes

Meta-regression was performed to investigate the potential effect of the time period in which each study was undertaken on between-study heterogeneity in the causes, management, and outcomes of heart failure.

**Figure 14:**
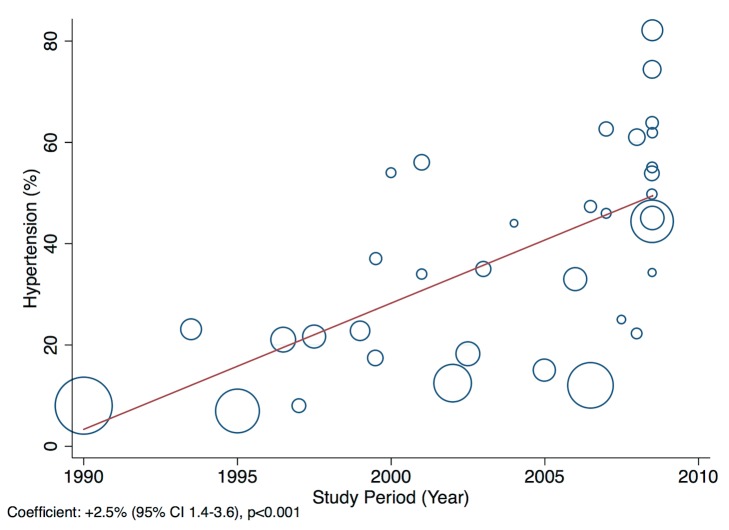
Meta-regression of hypertension against study period.

**Figure 15:**
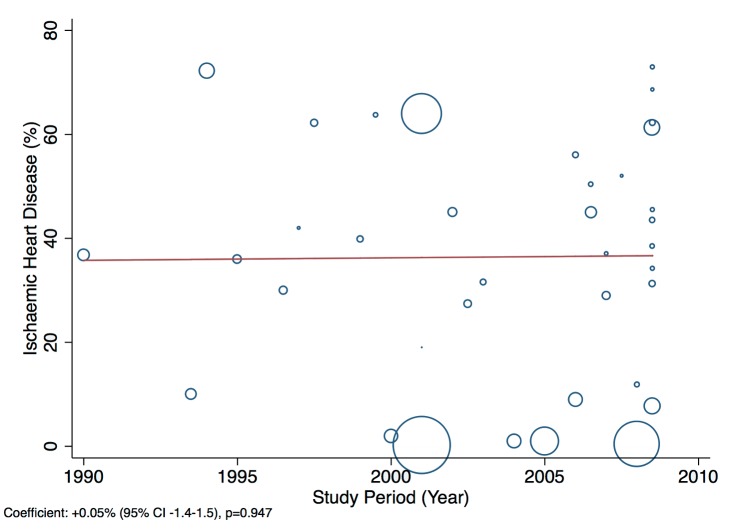
Meta-regression of ischaemic heart disease against study period.

**Figure 16:**
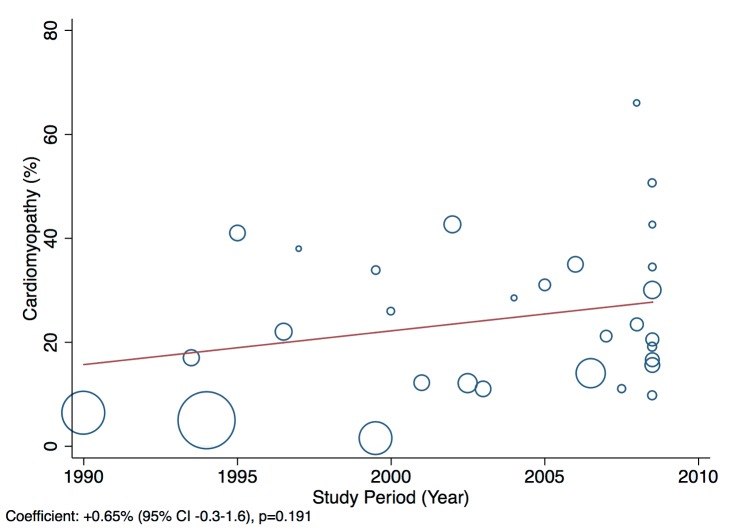
Meta-regression of cardiomyopathies against study period.

**Figure 17:**
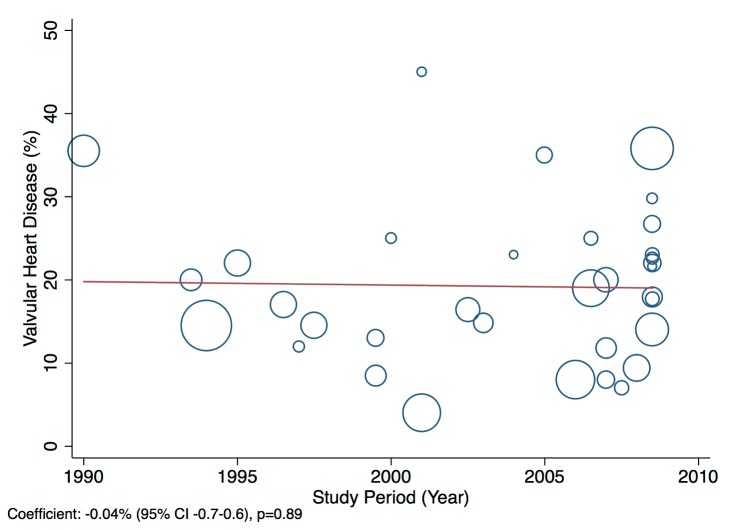
Meta-regression of valvular heart disease against study period.

A statistically significant effect was observed between the study time period and hypertension as a cause of heart failure, which rose by 2.5% per year (95% CI: 1.4%–3.6%, *p*<0.001) between 1990 and the late 2000s (Figure 14). There was no evidence to suggest that study time period had a significant effect on the other main causes of heart failure (IHD: 0.05%, 95% CI: −1.4% to 1.5%, *p*<0.95; cardiomyopathies: 0.65%, 95% CI: −0.3% to 1.6%, *p*<0.19; valvular heart disease: −0.04%, 95% CI: −0.7% to 0.6%, *p*<0.89) (Figures 15–17).

**Figure 18:**
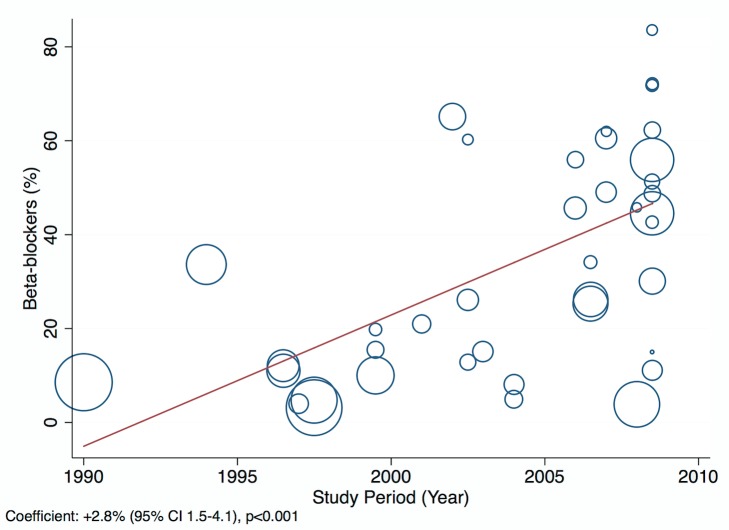
Meta-regression of beta-blocker use against study period.

**Figure 19:**
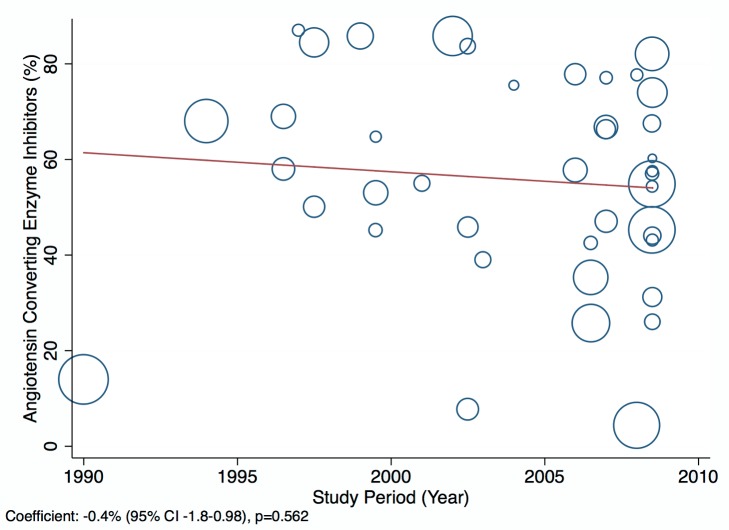
Meta-regression of angiotensin-converting enzyme inhibitor use against study period.

**Figure 20:**
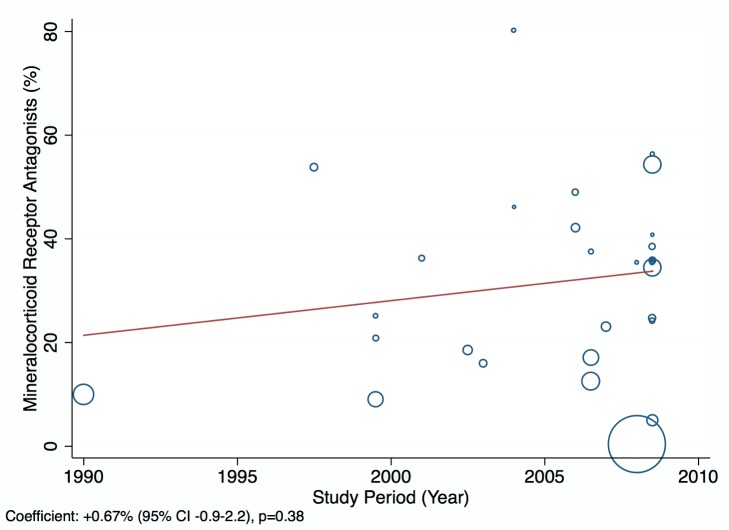
Meta-regression of mineralocorticoid receptor antagonist use against study period.

**Figure 21:**
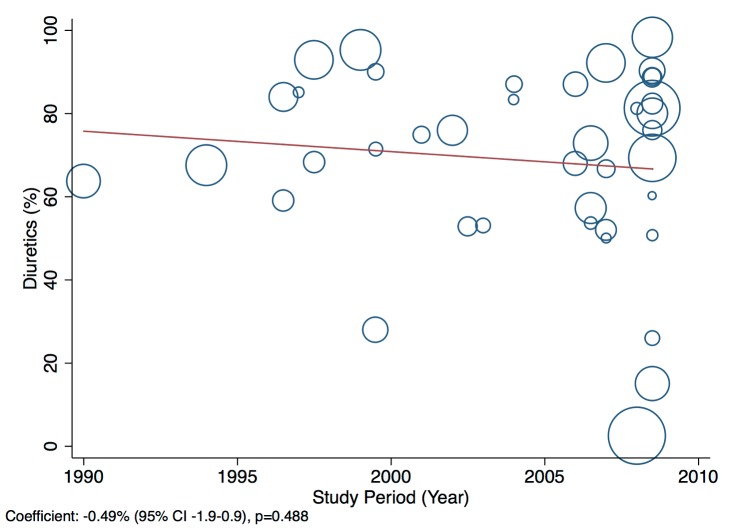
Meta-regression of diuretic use against study period.

The reported utilization rates for medical treatments of heart failure did not differ significantly over time, with the exception of beta-blockers, which showed an increase of 2.8% per year (95% CI: 1.5%–4.1%, *p*<0.001) (Figure 18). Corresponding figures were −0.4% per year (95% CI: −1.8% to 0.98%, *p* = 0.56) for ACEI use and 0.67% (95% CI: −0.9% to 2.2%, *p* = 0.38) for mineralocorticoid receptor antagonist use, with loop and/or thiazide diuretic use changing by −0.49% per year (95% CI: −1.9% to 0.9%, *p* = 0.49) (Figures 19–21).

**Figure 22:**
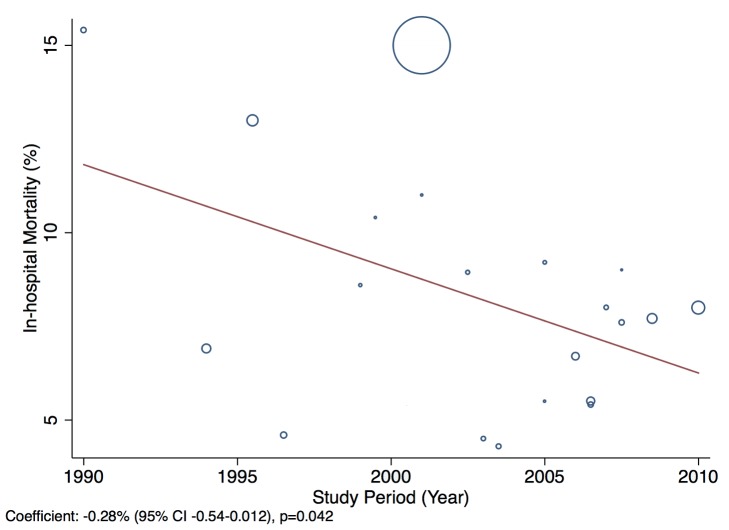
Meta-regression of in-hospital mortality rates against study period.

There was also some evidence to suggest in-hospital mortality rate declined by 0.28% per year between 1990 and 2010 (95% CI: −0.54% to −0.012%, *p* = 0.042) (Figure 22).

## Discussion

Our study presents, to our knowledge, the most comprehensive review to date and the first pooled analysis of the burden of heart failure in LMICs worldwide, collating data on over 230,000 episodes from 31 countries, with representation from all world regions. We found that heart failure is already a major burden to populations and health services in LMICs, where it makes up an average of 2.2% of hospital admissions, affecting more men than women. Reflecting the broad range of countries included and their differing levels of socio-economic development, there are wide variations in patient characteristics and the causes of heart failure and its management. Nonetheless, noticeable similarities can be discerned both between the included LMICs themselves and between these LMICs and HICs.

Across all LMICs from which data were available, the mean age of patients was 63 y, which is over a decade younger than in studies from HICs [76],[77]. The observed differences in age between the countries correlated strongly with the differences in HDI across them. Alongside this is the graded rise in the mean age of patients from represented low-income, low-middle-income, and upper-middle-income countries, from 50 y in the former to 60 y and 70 y, respectively. Thus, the age of presentation in upper-middle-income countries comes close to that in HICs (70 y in the EuroHeart Failure Survey II across 30 countries in Europe [76] and 72 y in ADHERE in the US [77]).

Substantial inter-regional variation is present in the causes ascribed to individual cases of heart failure. Heart failure is a syndrome made up of a constellation of signs and symptoms, with additional features present on further investigation. Given that a number of its aetiological underpinnings are often potential co-morbidities, disentangling one from the other is fraught with challenges, particularly in low-resource environments without recourse to a broad range of investigative tools [31]. Although 80% of studies from the Americas, Western Pacific, and Europe reporting aetiologies for heart failure documented the use of additional investigative tools, only 50% of studies from Africa did so. Nevertheless, our results are broadly consistent with the patterns of risk factors reported by Khatibzadeh and colleagues in their recent review of the worldwide risk factors for heart failure [78], as well as those of the Global Burden of Disease Study [10]. It is of note that preventable non-communicable diseases, in particular IHD and hypertension, are responsible for the large majority of cases worldwide.

Current guidelines worldwide stress the importance of ACEIs, beta-blockers, and mineralocorticoid receptor antagonists in the management of heart failure with reduced LVEF, with loop/thiazide diuretics given for symptom relief. Across the 29 studies from which management data were available, few studies reported the LVEF of patients, and fewer still separated data by LVEF. Overall mean LVEF was 40%: 38% amongst inpatients and 48% amongst those in non-acute settings. Consequently, it is not possible to make strong conclusions about the adherence of practice to evidence-based practices worldwide, but it is evident that management diverges considerably between regions and remains suboptimal on average. Data from the EuroHeart Failure Survey II of 30 high-income European countries also demonstrated poor medical management [76]. In this study, the mean LVEF of patients was 38%, and just over one-third of patients had a LVEF>45% [76]. Here, 71% of individuals were prescribed ACEIs, 48% a mineralocorticoid receptor antagonist, and 61% a beta-blocker at discharge [76]. The corresponding figures across our dataset are 57%, 32%, and 34%, respectively.

Across represented LMICs, patients admitted with heart failure had a poorer immediate prognosis than those in many HICs. However, as is the case for HICs, the estimates from LMICs varied substantially, although we found the difference between the two outlying regions in terms of prognosis, the Americas and South East Asia, was not statistically significant (*p* = 0.27). On average, the in-hospital mortality rate was 8.3% in LMICs, compared to 6.7% in the EuroHeart Failure II Survey [76] and 4% in ADHERE in the US [77]. Such differences, and the wide heterogeneity both within LMICs and between LMICs and HICs, may be due to different thresholds for hospitalisation or differences in patient characteristics, treatment strategies, or hospital characteristics. Reports of outcomes after hospital discharge were available from some studies, and these were more comparable to estimates from HICs [4],[6],[19],[42],[47].

Remarkable regional variation exists in the incidence of heart failure admissions to hospital. Of particular note is the low rate of reported admissions for heart failure in India and Iran. Unpublished data from India, based on the hospital billing codes assigned to patients from a sample of just under 1,551,410 admissions, showed an incidence of 0.37% [69]. Similarly, 0.3% of all hospital admissions were attributed to heart failure in a registry of over 80,000 hospitalisations across a number of hospitals in Iran (S. Rahimzadeh, F. Farzadfar F, and M. Ghaziani, unpublished data). These figures are an order of magnitude smaller than what is reported in HICs. There are several possible reasons for this observation. For example, it may be that in these countries, hospitals are still largely used for procedure-related activities, as opposed to pure medical management. In such a setting, treatment of medical conditions, such as heart failure, is much more likely to take place in the outpatient setting, for which data from India and Iran are lacking. Overall, the population-level incidence and prevalence of heart failure, despite its significance and dominance amongst cardiovascular diseases presenting to hospitals worldwide, remains largely unknown. Similarly, few data regarding the direct and indirect costs of heart failure are available in LMICs, information that is vital in understanding and measuring the value of different health service configurations and novel interventions.

This review collates data over a time period of almost 20 y, which may be one explanation for the degree of heterogeneity in results between studies. However, when study period was analysed using meta-regression against the causes, management, and outcomes of heart failure, only three statistically significant effects were found. These included a rising percentage of patients in whom hypertension was reported as a contributing cause of heart failure, an increasing trend in the reported prescription of beta-blockers over time, and a substantial decline in in-hospital death rates (see Figures 14, 18, and 22). Although these associations are plausible and—in case of beta-blocker use and mortality rates—encouraging, they should be interpreted cautiously because of the potential for confounding.

### Limitations

The data included are derived from a heterogeneous group of studies that set out with differing research goals. Variation in the methodologies used, particularly in methods of standardising the diagnosis and assessment of heart failure, may impact on some of the findings. These factors likely explain the high estimates of between-study variation that we found. Such variation may lead to underestimation of the true prevalence of heart failure, as well as inaccuracies in the causes ascribed to cases of heart failure. Our study includes individuals from three groups: those with their first presentation with acute heart failure, those with acute decompensation of chronic heart failure, and those with stable chronic heart failure seen in the outpatient clinic setting. Differences between healthcare systems may mean that the characteristics of patients seen in various settings may differ between countries, whilst adherence to gold-standard management may be more common amongst those with stable chronic heart failure seen in outpatient settings staffed by cardiologists than amongst those with acute heart failure treated in hospitals staffed by general internal physicians. In analysing these patients we have focussed on the evidence-based medical management methods common to all three groups. Combining data from 1995 to 2014, this study summarises management techniques over an almost 20-y period, an approach that may underestimate adherence to current management standards. However, when evaluated with meta-regression, the heterogeneity in a management variable was rarely found to be explained by changes over time. Another limitation of our study is that our data are derived from studies conducted for the most part in urban tertiary referral centres, which may not reflect the broader picture of heart failure in other hospitals and the community. Finally, despite the large number of studies included, information from some regions and for some outcomes was limited. In countries where few data are available, these results may not be truly reflective of the population and should therefore be interpreted as only a guide to the true prevalence, causes, and management of heart failure.

### Conclusion

This review shows that heart failure places a considerable burden on health systems in LMICs, and affects a wide demographic profile of patients in these countries. Non-communicable diseases dominate the causes of heart failure across LMICs, although infectious valvular diseases and cardiomyopathies continue to impose a significant burden. Together, this suggests a double burden of communicable and non-communicable diseases for countries in the midst of epidemiological transition. In addition, we have identified high in-hospital mortality and wide variation and significant suboptimal use of pharmacological therapies. Further population-level studies, with clear case and outcome definitions, are needed for a more accurate assessment of heart failure in LMICs.

## Supporting Information

Protocol S1 **Study protocol: systematic review of the burden of heart failure in low- and middle-income countries.**
(PDF)

Checklist S1 **PRISMA 2009 checklist.**
(DOC)

## Abbreviations

ACEI: angiotensin-converting enzyme inhibitor
ADHERE: Acute Decompensated Heart Failure Registry
CI: confidence interval
HDI: human development index
HICs: high-income countries
IHD: ischaemic heart disease
I PREFER: Identification of Patients with Heart Failure and Preserved Systolic Function
LMICs: low- and middle-income countries
LVEF: left ventricular ejection fraction
