# Supplementary material for: Heart Failure Care in Low- and Middle-Income Countries: A Systematic Review and Meta-Analysis
Source: PLoS Med. 2014 Aug 12;11(8):e1001699. doi: 10.1371/journal.pmed.1001699 (PMC4130667; doi:10.1371/journal.pmed.1001699)
Supplement: Protocol S1 — Study protocol: systematic review of the burden of heart failure in low- and middle-income countries. (PDF) [file pmed.1001699.s001.pdf]

Bennett et al. Systematic Reviews 2012, 1:59
http://www.systematicreviewsjournal.com/content/1/1/59

SYSTEMATIC REVIEWS

PROTOCOL Open Access

Study protocol: systematic review of the burden of heart failure in low- and middle-income countries

Derrick A Bennett[1], Thomas K Eliasz[2], Anna Forbes[2], Alastair Kiszely[2], Rajit Khosla[2], Tatjana Petrinic[3], Devarsetty Praveen[4,5], Roohi Shrivastava[2], Du Xin[6], Anushka Patel[4,5], Stephen MacMahon[2,4] and Kazem Rahimi[2,7*]

Abstract

Background: Setting priorities for the prevention and management of heart failure requires an empirical understanding of the pattern of disease burden. We aim to describe the methods for a systematic review of the literature on burden of heart failure in low- and middle-income countries (LMIC) and how this information will be synthesized to produce useful estimates that can inform policy and practice.

Methods: We will conduct a comprehensive search strategy for articles published between 1995 and April 2012 related to incidence, prevalence and treatment of heart failure in LMIC. Populations will be coded as urban, rural, or combined and studies classified as national, sub-national, healthcare system-based, or community level. Details from eligible studies will be extracted independently by two reviewers using a pre-designed data extraction form that will cover information on demographics, diagnostic criteria including disease incidence and prevalence, medical history, medication history, and hospital- or community-based management and outcomes. We will assess the reporting and methodological quality of the included studies and conduct a quantitative summary of reported outcomes where appropriate.

Discussion: Currently, there are important gaps in our knowledge on the burden of heart failure in LMIC and this systematic review aims to provide useful information that improves our knowledge in this field. Results are expected to be publicly available in early 2013.

Keywords: Heart failure, Incidence, Meta-analysis, Prevalence, Treatment

Background

The increasing prevalence of heart failure is a recognized major public health issue in most high-income countries [1,2]. For instance, in the UK, about 1% of the population suffers from chronic heart failure but the prevalence increases rapidly with age, affecting about 7% of the population aged 75 years or more [3]. Heart failure is already one of the leading causes of admission to, and bed occupancy in, UK hospitals, surpassing all other cardiac conditions [3]. Incidence and prevalence of heart failure in other developed countries are similar to those in the UK, rendering it a great burden to health services and patients in high-income countries [4,5].

More recently, cardiovascular disease has become one of the major causes of premature death and disability in low- and middle-income countries (LMIC). While this is expected to lead to a growing burden of heart failure in such countries, there is little systematic data about incidence, prevalence, underlying causes, and management of heart failure in these regions [6-8]. For example, a review conducted in 2000 found no published population-based studies of heart failure in the developing world and only very limited information from case series and hospital-based studies [6].

We aim to fill this gap in knowledge by conducting a systematic review of the contemporary literature on the 'burden' of heart failure from less developed countries.

* Correspondence: kazem.rahimi@georgecentre.ox.ac.uk
[2]George Centre for Healthcare Innovation, University of Oxford, Richard Doll Building, Old Road Campus, Oxford OX3 7LF, UK
[7]Department of Cardiovascular Medicine, University of Oxford, Oxford, UK
Full list of author information is available at the end of the article

BioMed Central

© 2012 Bennett et al.; licensee BioMed Central Ltd. This is an Open Access article distributed under the terms of the Creative Commons Attribution License (http://creativecommons.org/licenses/by/2.0), which permits unrestricted use, distribution, and reproduction in any medium, provided the original work is properly cited.

This will update previous reviews in this area [6] and go beyond other recent reports, which focused on regional variation in heart failure epidemiology worldwide [5].

Objectives

The overall aim of this paper is to present a transparent process for how the information will be collected on the burden of heart failure in LMIC. This will be based on explicit definitions and summarize the techniques that will be used to maximize the validity of these measurements by addressing bias, confounding, and missing data. More specifically, we aim to: describe the key research questions that this review will address; document our systematic literature search strategy; describe criteria for inclusion or exclusion of studies and other data sources identified in the review; describe study coding procedures, data categorizations, and study quality measures for the systematic review; and describe statistical procedures for the quantitative analysis of data from eligible studies.

Methods

Diagnosis of heart failure and the role of diagnostic testing

Heart failure is not a distinct disease but a syndrome with several potential underlying causes and precipitants, such as myocardial infarction, valve disease, or non-cardiac conditions. Once diagnosis has been established, further investigations are usually required to elicit the underlying cause of the heart failure. Commonly, diagnosis is based on a combination of clinical examination, electrocardiogram, chest X-ray, echocardiography, and blood concentrations of natriuretic peptides (Brain Natriuretic Peptide or N-terminal pro-B type natriuretic peptide levels) [7]. The availability of these tests and the approach to diagnosis of heart failure are likely to be highly variable in different settings. In the current study, we will not restrict studies to a particular case definition. Instead we will record the diagnostic criteria used for case definition in each study and will then assess its quality according to international guidelines for diagnosis of heart failure [7].

Research questions

Our literature review aims to address the following research questions. In LMIC,

1. What is the contemporary incidence and prevalence of heart failure?
2. What are the common causes or determinants of heart failure?
3. What is the burden of heart failure to health services, in terms of proportion of admissions to hospital, length of stay and resource utilization?
4. How are patients with heart failure diagnosed and managed?

These questions are highly relevant to policy makers and service providers. However, addressing these questions requires reviewing different types of literature. Information about incidence and prevalence of heart failure can be extracted from population-based cohort or cross-sectional studies, whereas resource utilization and management of heart failure patients is usually reported in hospital-based or provider-based studies. The need for consideration of different study designs and settings is reflected in our search strategy and eligibility criteria.

Study eligibility

Studies among any population(s) from throughout the world are potentially eligible for inclusion in this overview if they satisfy all the following criteria: population-based cohort study, or registries and hospital databases, or cross-sectional studies such as census data; sample size restricted to more than 100 cases of heart failure as defined by the authors; for population-based cohort or cross-sectional studies: reports on incidence or prevalence (by age, gender and so on.); for hospital-based cohort or cross-sectional studies: reports on proportion of heart failure admissions to total admissions (prevalence), causes or underlying reasons, death and readmission rates (for example, in hospital, 30-day, or annual rates), length of stay, and/or management (such as prescription rates); geographic regions confined to LMICs in Sub-Saharan Africa, Mediterranean region (excluding Europe), Central and Latin America, South, South East, South West, and Central Asia, and China.

Studies confined to subgroups of patients with heart failure (for example, with dilated cardiomyopathy or heart failure as a complication of acute myocardial infarction) will be excluded, as will studies that do not include a representative sample of patients (for example, studies that selected people referred to a echocardiography department). We aim to report information at individual country or regional level. Investigators of multinational studies that have not reported findings by each country or region will be contacted for provision of region- or country-specific data.

Search strategy for identification of relevant studies

As we are primarily interested in the contemporary literature on this topic, we will search MEDLINE, Embase, Global Health Database (formerly CAB abstracts), and World Health Organization regional databases) for articles published between 1995 and April 2012 with any of the subject terms “heart failure” and “cardiomyopathies” or any of the following terms in the title or abstract: ‘heart failure’, ‘cardiomyopath*’, ‘cardiac failure’ AND ‘incidence’, ‘prevalence’, ‘cause*’, ‘etiology’, ‘aetiology’, ‘epidemiolog*’, ‘burden’, ‘management’, ‘treatment’, ‘prevent*’, ‘population based’, ‘community’,

'trends', 'survey', 'surveillance', 'mortality', 'morbidity', 'fatalit*' or 'attack rate'. The search will further be limited to regions outside Europe and North America and to human studies with no language restriction. We will also scrutinize the reference lists of study reports and review articles, and inquire among collaborators and colleagues.

Screening of abstracts

After removing duplicate reports, two reviewers will independently screen all titles and abstracts for their potential eligibility as described previously. Specifically, titles and abstracts are included if they indicated that it was a population-based or hospital-based study reporting any relevant information on incidence, prevalence, case-fatality or mortality, or management and outcomes of patients with heart failure in a well-specified population. Studies might be based on cohorts, cross-sectional surveys, disease registry, hospital surveys, or notification data. The list of all titles and abstracts identified will provide a pool of the epidemiological evidence-base for heart failure in LMIC. In the next stage, members of the review team will identify all studies that potentially meet the eligibility criteria that provided the most relevant data for the measures of interests. Full reports of these citations will be obtained for further assessment of the study for inclusion in the systematic review. In the third stage, we will categorize the available information by region and will contact local experts for any additional information that they may be aware of. If there are reports from a country or region covering multiple time-points, we aim to use either the most up-to-date or the most robust information available.

Data extraction

Details from eligible studies will be extracted independently by two reviewers using a pre-designed data extraction form. Where the data that is extracted differ between assessors, the discrepancy will be resolved by consensus and, when necessary, additional information will be sought from the authors of the studies. Where differences in opinion still exist, a third party will be consulted. The data extraction form will include the following items:

General information: Name of study, country, or region where study was conducted, year of publication, journal, language of publication, contact address of corresponding author.
Reason for exclusion: Irrelevant geographic region, less than 100 cases, no relevant information about disease burden, management, or outcomes.
Population characteristics: Demographic details (for the overall population and for the cases separately), for example, age, gender, and ethnicity.
Case definition and description: Diagnostic criteria and investigations used, including any details on specific measures such as laboratory information (for example, biomarkers), electrocardiogram, chest X-ray, or echocardiographic findings.
Causes of heart failure and any other relevant co-morbidities: History of coronary artery disease, hypertension, diabetes, valve disease, rheumatic heart disease, atrial fibrillation, renal failure, cardiomyopathy, chronic obstructive lung disease, or smoking.
Medication history: Percentage use of aspirin, warfarin, diuretics, beta-blockers, angiotensin-converting enzyme-inhibitors, or angiotensin-receptor blockers, percentage of other relevant medications or device therapies.
Hospital-based study outcomes: Proportion of cases to the total number of population included in the study (prevalence), duration of stay, mortality rate, readmission rate, prevalence of medications at discharge from hospital.
Community-based study outcomes: Incidence, prevalence, mortality rate, prevalence of medication use in the community.
Methodological information: Study design, urban or rural population, completeness of case ascertainment, representativeness, validity of case ascertainment, reliability of outcome data.

Quality assessment

We will separate out methodological quality from general reporting quality as it is important to clarify and differentiate between quality of reporting and the quality of what was actually done (that is, a study could be well reported but have methodological limitations or vice versa) [9]. As a variety of study types and data sources are likely to be eligible from the heart failure literature, information will be recorded on the appropriateness of the particular study design to estimate relevant disease parameters; whether the data are representative of the population; and methodological quality and completeness of data reported. Methodological quality refers to the extent to which specific aspects of a study or data source can be shown to protect against systematic bias, non-systematic bias, and inferential error for the outcomes of interest.

We intend to assess the general reporting quality of the study using a selection of items derived from those included in the Strengthening the Reporting of Observational Epidemiology statement for assessing the quality of observational studies [10]. We will assess specific items of reporting quality, such as use of a clear definition of heart failure; that the design and methods are appropriate for the research question; that the sample size is adequate for robust estimates; that the denominator data are reliable

and sources documented; that confidence intervals or variance estimates are reported (or can be derived from the information reported); that the sources of potential bias are adequately controlled; and that the study limitations that can influence interpretation of findings are identified.

The collection of this general reporting quality information as well as methodologically-specific quality information enables the exploration of possible sources of heterogeneity and will also be used to conduct sensitivity analyses to quantify the magnitude of possible bias in study estimates based on particular characteristics of the study. Sensitivity analyses will be based on stratification by individual items of methodological quality or (where appropriate) individual items of general reporting quality to assess the robustness of the findings.

Quantitative synthesis of individual estimates

It is envisaged that many of the studies will report their results as either numbers of participants or as a percentage of the total population for many of the outcomes of interest (for example, incident heart failure, co-morbidities, types of investigations performed, and discharge medication). It should be possible to derive appropriate point estimates and measures of variability from this type of information if an appropriate denominator is available. For continuous outcomes, such as length of stay, if a standard deviation is not reported, it is possible to derive an estimate of the variability based on the range [11].

The individual study estimates extracted will be combined using inverse-variance weighting techniques to provide information on the relevant estimates of incidence, prevalence, and other relevant outcomes (if this is appropriate). This implies that larger studies will be given more weight in the analyses when producing an overall pooled estimate for a particular low- or middle-income country. This approach is more robust to small study biases (of which publication bias is just one of aspect of this umbrella term) [12]. We will investigate potential sources of heterogeneity (as assessed by the standard Cochrane Q statistics and the I-squared statistic that can be derived from the Cochrane's Q), related to both methodological and clinical characteristics of the studies [13]. We will also aim to investigate whether there is effect modification in particular subgroups such as by age, sex, region, and ethnicity if we have sufficient information available [14].

Preliminary report

Figure 1 summarizes the retrieval and selection process for studies. The combined searches yielded 3,275 reports, of

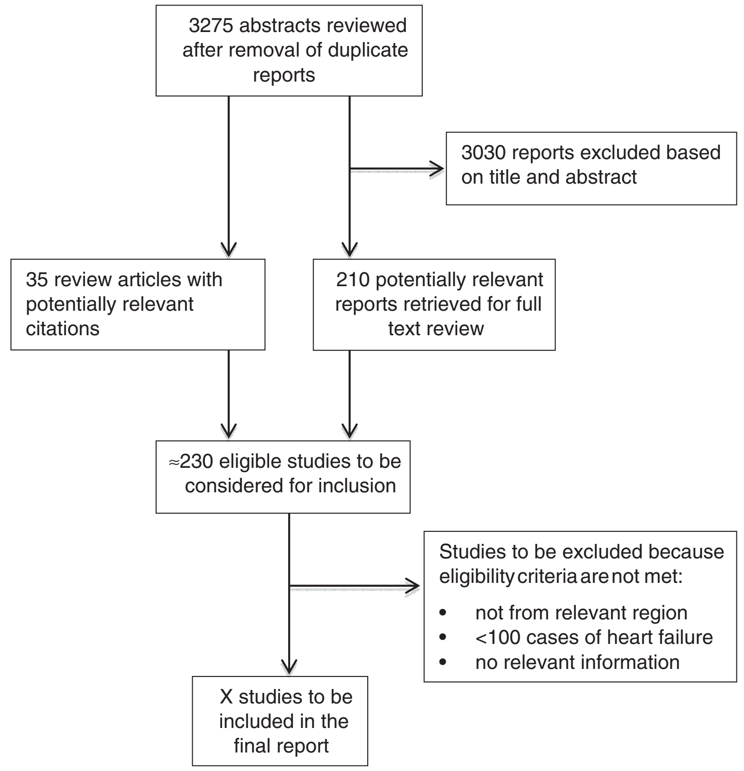

Figure 1 Search retrieval process.

which 3,030 were excluded on the basis of titles and abstracts. Full text articles are currently being retrieved for 210 potentially relevant studies. In addition, 35 reviews or commentary articles relating to heart failure burden or management in LMIC have been identified. These articles are currently being reviewed for any potentially relevant reference that they may contain. An electronic data extraction form has been piloted (the form can be obtained from the corresponding author upon request), and full text data extraction will begin once all potentially full text articles have been retrieved.

Discussion

Ageing populations with an increasing prevalence of heart failure place a growing burden on health systems in many parts of the world. Crude estimates based on limited data suggest that heart failure may have become the most rapidly growing cardiovascular condition worldwide in terms of the absolute number of cases diagnosed each year [15]. Heart failure is associated with substantial morbidity and mortality [1,2] and is one of the leading causes of admission to hospital in many high-income countries [15]. However, there are currently important gaps in our knowledge on the incidence and prevalence of heart failure and management patterns for such patients in countries outside North America and Europe [8]. Setting priorities in service delivery for the prevention and treatment of heart failure requires an empirical understanding of the pattern of disease burden. This systematic review aims to provide useful information that improves our knowledge in this field. We expect this review to fill the gap in knowledge in burden of disease in LMIC [6] and to extend recent work in the areas of management practices and impact on health services [5]. This new information should be able to form the basis of an evidence-base for policy and practice in these regions. Currently, we have screened over 3,000 abstracts and identified about 200 potentially relevant studies, which will be subject to full text review. We expect data extraction to be completed by the end of 2012. Preliminary analyses will be conducted by the end of the first quarter of 2013. Results are expected to be publicly available by mid-2013.

Competing interests

The authors declare that they have no competing interests.

Authors' contributions

KR and DB designed the study with input from DX, AP and SM. TKE, AF, AK, RK, DP and RS have led the strategy for abstract reviews and data extraction plans. TP conducted a scoping review of the literature and devised the final bibliographic search. KR and DB drafted the first version of the manuscript with significant input from all other authors. All authors have read and approved the final version of the manuscript.

Acknowledgements

KR and SM are supported by the Oxford Martin School and the Oxford NIHR Biomedical Research Centre.

Author details

[1]Clinical Trial Service Unit and Epidemiological Studies Unit, University of Oxford, Oxford, UK. [2]George Centre for Healthcare Innovation, University of Oxford, Richard Doll Building, Old Road Campus, Oxford OX3 7LF, UK. [3]Bodleian Healthcare Libraries, University of Oxford, Oxford, UK. [4]The George Institute for Global Health Australia, Sydney, Australia. [5]The George Institute for Global Health India, Hyderabad, India. [6]The George Institute for Global Health China, Beijing, China. [7]Department of Cardiovascular Medicine, University of Oxford, Oxford, UK.

Received: 19 July 2012 Accepted: 12 November 2012
Published: 29 November 2012

References

1. Roger VL, Weston SA, Redfield MM, Hellermann-Homan JP, Killian J, Yawn BP, Jacobsen SJ: **Trends in heart failure incidence and survival in a community-based population.** *JAMA* 2004, **292**(3):344–350.
2. Mehta PA, Dubrey SW, McIntyre HF, Walker DM, Hardman SMC, Sutton GC, McDonagh TA, Cowie MR: **Improving survival in the 6 months after diagnosis of heart failure in the past decade: population-based data from the UK.** *Heart* 2009, **95**(22):1851–1856.
3. Audit NHF: *National Institute for Cardiovascular Outcomes Research (NICOR), the Institute of Cardiovascular Science*. London: University College London; 2012 www.ucl.ac.uk/nicor/nicor/hfannualreport10-11.
4. Mosterd A, Hoes AW: **Clinical epidemiology of heart failure.** *Heart* 2007, **93**(9):1137–1146.
5. Geographic Variation in the Causes of Heart Failure: *A Systematic Review for the Global Burden of Disease Study*. Dubai: World Congress of Cardiology; 2012.
6. Mendez GF, Cowie MR: **The epidemiological features of heart failure in developing countries: a review of the literature.** *Int J Cardiol* 2001, **80**(2–3):213–219.
7. Dickstein K, Cohen-Solal A, Filippatos G, McMurray JJ, Ponikowski P, Poole-Wilson PA, Strömberg A, van Veldhuisen DJ, Atar D, Hoes AW, Keren A, Mebazaa A, Nieminen M, Priori SG, Swedberg K, ESC Committee for Practice Guidelines (CPG): **ESC guidelines for the diagnosis and treatment of acute and chronic heart failure 2008: the Task Force for the diagnosis and treatment of acute and chronic heart failure 2008 of the European Society of Cardiology. Developed in collaboration with the Heart Failure Association of the ESC (HFA) and endorsed by the European Society of Intensive Care Medicine (ESICM).** *Eur J Heart Fail* 2008, **10**(10):933–989.
8. Rahimi K, Patel A, MacMahon S: **Two decades of research on innovative models of care delivery for patients with heart failure: the end or just the beginning?** *Arch Iran Med* 2012, **15**(7):439–445.
9. Shamliyan T, Kane RL, Dickinson S: **A systematic review of tools used to assess the quality of observational studies that examine incidence or prevalence and risk factors for diseases.** *J Clin Epidemiol* 2010, **63**(10):1061–1070.
10. von Elm E, Altman DG, Egger M, Pocock SJ, Gøtzsche PC, Vandenbroucke JP, STROBE Initiative: **The Strengthening the Reporting of Observational Studies in Epidemiology (STROBE) statement: guidelines for reporting observational studies.** *PLoS Med* 2007, **4**(10):e296.
11. Hozo SP, Djulbegovic B, Hozo I: **Estimating the mean and variance from the median, range, and the size of a sample.** *BMC Med Res Methodol* 2005, **5**:13.
12. Sterne JA, Egger M, Smith GD: **Systematic reviews in health care: investigating and dealing with publication and other biases in meta-analysis.** *BMJ* 2001, **323**(7304):101–105.
13. Thompson SG: **Why sources of heterogeneity in meta-analysis should be investigated.** *BMJ* 1994, **309**(6965):1351–1355.
14. Higgins JP, Thompson SG: **Quantifying heterogeneity in a meta-analysis.** *Stat Med* 2002, **21**(11):1539–1558.
15. McMurray JJV, Stewart S: **The burden of heart failure.** *Eur Heart J Suppl* 2002, **4**(suppl D):D50–D58.

doi:10.1186/2046-4053-1-59
Cite this article as: Bennett *et al.*: Study protocol: systematic review of the burden of heart failure in low- and middle-income countries. *Systematic Reviews* 2012 **1**:59.
